# The effect of deformation of absorbing scatterers on Mie-type signatures in infrared microspectroscopy

**DOI:** 10.1038/s41598-021-84064-5

**Published:** 2021-02-25

**Authors:** Maren Anna Brandsrud, Reinhold Blümel, Johanne Heitmann Solheim, Achim Kohler

**Affiliations:** 1 Faculty of Science and Technology, Norwegian University of Life Sciences, Aas, Norway; 2 Department of Physics, Wesleyan University, Middletown, Connecticut USA

**Keywords:** Biophotonics, Biological physics, Micro-optics, Characterization and analytical techniques, Optical spectroscopy

## Abstract

Mie-type scattering features such as ripples (i.e., sharp shape-resonance peaks) and wiggles (i.e., broad oscillations), are frequently-observed scattering phenomena in infrared microspectroscopy of cells and tissues. They appear in general when the wavelength of electromagnetic radiation is of the same order as the size of the scatterer. By use of approximations to the Mie solutions for spheres, iterative algorithms have been developed to retrieve pure absorbance spectra. However, the question remains to what extent the Mie solutions, and approximations thereof, describe the extinction efficiency in practical situations where the shapes of scatterers deviate considerably from spheres. The aim of the current study is to investigate how deviations from a spherical scatterer can change the extinction properties of the scatterer in the context of chaos in wave systems. For this purpose, we investigate a chaotic scatterer and compare it with an elliptically shaped scatterer, which exhibits only regular scattering. We find that chaotic scattering has an accelerating effect on the disappearance of Mie ripples. We further show that the presence of absorption and the high numerical aperture of infrared microscopes does not explain the absence of ripples in most measurements of biological samples.

## Introduction

Infrared microspectroscopy is a technique that is well established in the biological sciences^1–4^. It allows investigating, e.g. cells and tissues, and bio-fluids in their native forms. For instance, using a synchrotron as the radiation source for performing infrared microspectroscopy, due to the brilliance of the synchrotron radiation, high-quality spectra can be obtained efficiently in the diffraction limit^5^. By the use of quantum cascade lasers (QCL) high quality images can be obtained, which have been reported to achieve a quality comparable or higher than images obtained when using synchrotron-based sources^6,7^. In infrared transmission microscopy of cells and tissues, purpose-built microscopes are used which allow to operate with radiation in the mid-infrared region. These systems are operated in two main modes. In one type of systems, infrared radiation is transmitted through cells and thin tissues, and the transmitted radiation is collected by a single-element detector after passing, e.g., through Schwarzschild optics. Alternatively, imaging systems are used where the transmitted radiation is mapped onto an imaging detector in forward direction. Since the size of the cell structures is of the same order as the wavelength of the infrared radiation, i.e., typically in the range of 2.5–$25\, {\upmu \rm m}$, infrared spectra of cells and tissues exhibit a variety of scattering phenomena^8^. The most intriguing scattering phenomena that have been observed are so-called Mie-type scattering phenomena^9–11^ and have been studied intensively in the literature during recent years^9–16^.

Any type of scattering poses a challenge for the interpretation of absorbance spectra, since scattering contributes to the extinction and is not easily separated from true absorption. Problems of this type are universally encountered whenever absorbance spectra of finite-sized samples have to be analyzed (see, e.g.,^9,17^). Therefore the topics and problems discussed in this paper are of general significance and wide applicability. Mie resonances, e.g., can either mimic or severely distort chemical absorption bands^11,18^. However, current correction algorithms are based on the assumption of spherical samples, an assumption that is often not justify in the case of biological cells or tissues. Therefore, the central point of our paper is to investigate how the deformation of (absorbing) biological samples influences and distorts Mie-type signatures.

Since Mie-type scattering occurs in spherical- and nearly-spherical shaped scatterers, strictly speaking, the term “Mie scattering” applies only to spherical scatterers. In this paper, for convenience, we will use the term Mie scattering also in the context of deformed scatterers. While the Mie theory is an exact theory and valid for any wavelength regime, size and refractive index of the sphere, strong and rich Mie scattering effects arise when the size of the scatterer is of the order of the wavelength of the incident light. Therefore, when using the term Mie scattering, we often refer specifically to this regime. When the size of the scatterer is of the same order as the wavelength employed, neither the long-wavelength Rayleigh limit nor the geometric-optics, short-wavelength ray limit, yield accurate results. The scattering of electromagnetic radiation at an ideal dielectric sphere was solved analytically by Gustav Mie already in 1908^19^. In Mie scattering, two main scatter contributions appear in the infrared spectra of spherically-shaped particles: (i) Broad oscillatory structures, so-called wiggles, which are due to the interference of the undisturbed, incoming radiation with the scattered radiation. (ii) Sharp scattering features, called ripples, which are due to standing waves, for instance, whispering gallery modes that are electromagnetic resonance structures trapped inside of the spherical scatterer. The broad oscillatory structures are well described by an approximation formula developed by van de Hulst^20^, which has been used extensively to establish algorithms that allow retrieving pure absorbance spectra^10,12,16^.

The broad Mie wiggles are generally observed in any type of infrared microscopy of cells and tissues. Pre-processing approaches have been developed to remove Mie wiggles from infrared spectra with the goal of obtaining pure chemical absorbance spectra^10^. Newer and more advanced approaches deal even with cases where strong dispersive effects due to absorption resonances are present in spectra^16^. While wiggles are omni-present in infrared spectra of cells and tissues, sharp ripples are rarely observed. They are observed mainly in cases where the scatterers represent perfect spheres such as PMMA spheres^11,21^, or shapes that are close to perfect spheres, such as pollen^22,23^. In most other infrared spectra of cells and tissues, the ripples are absent, while the wiggles are present.

Both ripples and wiggles are related to shape characteristics^24^. However, while wiggles are due to a robust interference process that does not rely on the intricacies of resonances, ripples, due to delicate resonance processes, were shown to be affected by absorption^23,25^. Ripples, if present, and wiggles are both strongly influencing the apparent absorbance spectra. It is known that they can lead to non-Beer-Lambert absorption behavior in the infrared spectroscopy of cells and tissues^9^. For instance, chemical absorption lines may be affected by ripples, since ripples are caused by resonant enhancement of the electric field in the sphere. It is also clear that, because they are shape resonances, the strengths of the ripples may depend strongly on the actual shape of the scatterer. Rasskazov et al. studied the effect of clusters of spheres on the extinction efficiency and the FT-IR spectra^26^. In addition, Davis et al. have evaluated the extinction efficiency of planar films and cylinders^27–29^. Sinusoidal oscillations in the simulated absorbance spectra of cylinders (toluene fibers) were also observed in^29^. However whether they are due to wiggles, ripples, or instrument properties cannot be ascertained without further analysis. In any case, the purpose of our paper is not to establish the presence of wiggles and ripples in the absorbance spectra of spheres or cylinders, which is a known property of these scatterers (see, e.g.,^11,20–25^), but to investigate how these features react to deformation, absorption, and numerical aperture for isolated spheres and quasi-spherical objects.

The sphere is one example of an exceedingly small class of systems for which analytical scattering solutions are known. Therefore, the sphere and its Mie solutions are frequently used as the starting point for the interpretation of infrared spectra. However, most scatterers occurring in nature, in particular in biology, are deformed in such a way that no analytical scattering solutions exist. These scatterers, any generically shaped biological cell amongst them, have no analytical scattering solutions and are said to be nonintegrable. Typically, nonintegrable shapes lead to chaotic scattering^30–32^. Therefore, chaotic scattering of biological cells and tissues is the rule rather than the exception. In the context of chaos in wave systems, it was shown that the presence of chaotic scattering behavior may enhance absorption properties of a scatterer considerably^33^. Small deviations from perfect spherical scatterers that involve changes in the shape or the refractive index of different parts of the sphere, may easily lead to a transition between regular and chaotic scattering^33^. The aim of the current study is to investigate how ripples and wiggles, observed in the scattering of cells and tissues, are influenced by the shape of the scatterer and the actual absorption properties of the scatterer. A deeper understanding of shape and absorption properties may help to improve Mie-scatter correction algorithms. For this purpose, we investigate a scatterer that is shaped like a Bunimovich billiard^34^. In the context of thin film solar cells, the Bunimovich billiard has been used as a model system for studying the solutions of the Laplace equation with boundary conditions that lead to chaotic behavior^33^. In most cases, the Bunimovich billiard has been studied as a bounded system, where a particle can move freely inside the Bunimovich billiard, but is not allowed to leave the billiard. In contrast, we use a dielectric Bunimovich billiard^35^ as a model for a chaotic scatter system to compare the scattering at a system without chaos, i.e., a spherical scatterer, with the scattering at a chaotic system, i.e., the stadium-shaped Bunimovich billiard^34,35^. In the case of the scatter system, we use different refractive indices inside and outside of the Bunimovich billiard and solve the corresponding electromagnetic or ray problem. The Bunimovich-billiard shaped scatterer has been shown to be chaotic^35^, and it allows to study the gradual transition between a regular scatterer (sphere) and a chaotic scatterer (billiard). The Bunimovich-billiard shaped scatterer^34^ is a ray-splitting system. Chaotic ray-splitting billiard systems have been investigated in the field of quantum chaos^36–39^. They provide model systems for investigating implications of interfaces between two dielectric media on the wave and ray dynamics of such systems. For completeness we also investigated, in addition to the chaotic Bunimovich-billiard shaped scatterer, the effects of deformation into an integrable scatterer, i.e., a scatterer which does not show a transition to chaotic scattering as a function of deformation. Comparing the two situations, we find that under increased deformation, chaotic scattering accelerates the destruction of shape-resonance phenomena, such as, e.g., Mie ripples in absorbance spectra, while resonance phenomena tend to be more robust under deformation in the case of integrable deformations, such as deformation into ellipsoids. This has important consequences, e.g., for infrared microspectroscopy: The effects of shape resonances, such as Mie ripples, may be important in the analysis of absorbance spectra of quasi-spherical/ellipsoidal samples, such as pollen and PMMA spheres, and may lead to serious errors in the analysis of spectra if not properly taken into account. However, in general, biological cells and tissues have structures that are very different from perfect spheres and ellipsoids, and in these cases, in addition to absorption, the effects of shape resonances are suppressed due to chaotic scattering.

In order to evaluate what causes the disappearance of ripples, three mechanisms are investigated. Our main study is to investigate how the wiggle and ripple structures are affected by a transition into a non-spherical shape. Further, we investigate how absorption affects the ripples and wiggles. We also evaluate how the numerical aperture of an infrared microscope and its size affects the extinction efficiency. Details of the latter two investigations can be found in the Supplementary Materials.

## Methods

### Infrared transmission measurements

**Figure 1:**
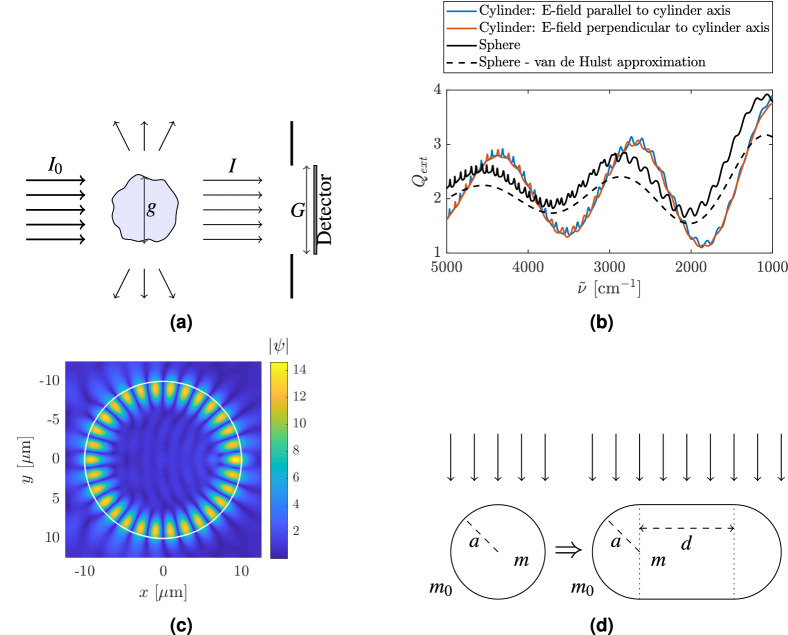
(**a**) Infrared radiation of intensity $I_0$ is sent towards a sample with geometrical cross section *g*. Part of the radiation is scattered off or absorbed by the sample. The radiation that is transmitted through the sample has an intensity of *I* and hits a detector with a geometrical cross section equal to *G*. (**b**) Extinction efficiency found from Mie Theory, $Q_{ext}$, and the van de Hulst approximation for $Q_{ext}$ for a sphere of radius $10\,\upmu \rm m$ and a refractive index of 1.3 (black line). The blue and the red lines indicate the exact extinction efficiency (described by Mie Theory) for an infinite cylinder with different polarization The radius of the cylinder is $10\,\upmu \rm m$ and the refractive index is 1.3. (**c**) Norm of the wavefunction, equivalent with the norm of the electric field $\vec{E}$, in the case where the electric field is parallel to the cylinder axis. The scatterer is a disk of radius $10\,\upmu \rm m$ and has an index of refraction of 1.8. The incident radiation is a plane wave with wavenumber $1643.5\,$ $\hbox {cm}^{-1}$, an amplitude equal to one, and is propagating from the left. The wavenumber of the incident plane wave is chosen to coincide with the wavenumber of a ripple, i.e., a standing wave along the inside of the circumference of the disk-shaped scatterer. (**d**) Illustration of how the system evaluated in this work transforms from a disk with a radius *a*, that is kept constant, into a stadium of width $2a + d$ with increasing *d*. The refractive index of the scatterer is *m* and the refractive index of the surroundings is $m_0 = 1$. The system is an open system, i.e., light can enter the system from the outside and can leave the system to the outside. The light is incident from the top as indicated by the arrows.

Figure 1a illustrates a typical infrared transmission measurement. Infrared radiation of intensity $I_0$ is directed toward a sample and the transmitted radiation *I* hits the detector. The background intensity, $I_0$, is measured by moving the sample out of the beam. The absorbance *A* is obtained by

1 $$\begin{aligned} A = - \log _{10}{\left( \frac{I}{I_0} \right) }. \end{aligned}$$

In this paper we use the term *absorbance* solely for the dimensionless quantity *A* and the term *absorption* solely for the phenomenon of conversion of light quanta into heat. In an ideal measurement, the measured absorbance *A* consists solely of absorption bands caused by chemical bonds that absorb in the sample. The infrared spectrum is known to be a highly reproducible fingerprint of the biochemical composition of cells and tissues that are under investigation. However, in many cases scattering phenomena lead to scattering signatures in the absorbance spectrum *A* in addition to the chemical absorption signatures. When scattering signatures are mixed with chemical absorption features in the measured absorbance spectrum, we call the measured absorbance, *apparent absorbance*^16,23^. In the case of a perfectly spherical scatterer, the scattering can be described exactly by Mie Theory^20,23^. Examples of such samples are some biological samples and PMMA spheres^22,23^. In order to retrieve and estimate pure absorbance spectra from highly scatter-disturbed measured infrared microspectroscopic measurements of single cells and tissues, the Mie extinction extended multiplicative signal correction (ME-EMSC) algorithm can be applied^16^.

### Calculation of the extinction efficiency

The apparent absorbance is related to the extinction efficiency, $Q_{ext}$, by

2 $$\begin{aligned} A = -\log _{10} \left( 1 - \frac{g}{G} Q_{ext} \right) \approx \frac{1}{\ln (10)}\frac{g}{G} Q_{ext}, \end{aligned}$$

where *G* and *g*, as illustrated in Fig. 1a, are the geometrical cross sections of the detector and the sample, respectively, and the approximation is obtained by expanding the logarithm to first order and assuming that $G>> g$^22^. $Q_{ext}$, a dimensionless quantity, is used to describe the amount of radiation which is extinguished from the forward direction^20^, either by absorption or by scattering.

For a sphere, as illustrated by the black, solid line in Fig. 1b, $Q_{ext}$ can be computed exactly via Mie Theory^20^. The refractive index of 1.3, used in Fig. 1b, is approximately that of water, close to the refractive indexes of many biological samples^40,41^. As shown by the black lines in Fig. 1b, $Q_{ext}$ consists of long-range oscillations, called *wiggles* (dashed black line), well-described by the *van de Hulst approximation*^20^, and sharp, narrow resonance structures, called *ripples*. The ripples are due to standing waves, i.e., resonances, called *whispering gallery modes* (WGMs)^21^, characterized by a mode structure that is concentrated at the circumference of the spherical scatterer. A typical WGM is illustrated in Fig. 1c. While Fig. 1c shows a WGM for a circular cylinder, there is no qualitative difference between WGMs for spheres and cylinders.

In this work we are evaluating two-dimensional systems, or systems which are translationally invariant in the third dimension, i.e., right cylinders. Circular, right cylinders can also be described exactly by electromagnetic theory^20^ (see Supplementary Materials Sec. A for details). We have further restricted our work to the case where the propagation direction of the incoming plane wave is perpendicular to the cylinder axis. In addition to $Q_{ext}$ for a sphere, as discussed above, Fig. 1b shows the extinction efficiency $Q_{ext}$ for an infinite cylinder with the $\vec{E}$-field parallel to the cylinder axis (blue line) and the $\vec{E}$-field perpendicular to the cylinder axis (red line). Analytical expressions for $Q_{ext}$, and in addition for the $Q_{sca}$ (scattering efficiency) and $Q_{abs}$ (absorption efficiency), are given in the Supplementary Materials (Sec. A) and in^20^.

In the case where the electric field is parallel to the cylinder axis, the situation is equivalent to a two-dimensional scalar wave problem where a plane wave is propagating towards a circular scatterer, and an exact description of the field inside and outside the scatterer can be found. Details are given in the Supplementary Materials Sec. BB.

Figure. 1c shows the norm of the wavefunction for a circular cylinder which corresponds to a resonance (ripple) at 1643.5 $\hbox {cm}^{-1}$. As discussed above, this mode structure corresponds to a WGM and illustrates the tight connection between WGMs and ripples in $Q_{ext}$. A WGM is confined to the interior of a sphere or cylinder by total internal reflection. This explains why the intensity of a WGM or the visibility of a ripple depends on the refractive index contrast between the outside and the inside of the scatterer. If the difference of the refractive index between the outside and the inside is large, the leakage of radiation to the outside is small and the WGMs and associated ripples are strong. Because they depend on total internal reflection, WGMs can be associated with classical rays that bounce along the circumference of the scatterer. It is noteworthy that the rays cannot leave the cylinder since they always bounce off the circumference with an incident angle larger than the critical angle for total internal reflection. Therefore the rays cannot be reached from the outside of the scatterer. As indicated in Fig. 1b, WGMs (ripples) can also be found in cases of small refractive index contrast . But the well-recognizable patterns, as shown in Fig. 1c, are more distinct in cases with a large refractive indicex contrast between inside and outside of the scatterer.

While real cells are not perfectly spherically shaped, Mie extinction extended multiplicative scattering correction (ME-EMSC) has been proven useful for scatter corrections in many practical situations. The ME-EMSC algorithm uses the van de Hulst approximation for scattering off spheres^10,12,16^. A possible problem of this method is that the van de Hulst approximation predicts the wiggle structure of the scattering, but not the ripples^20,21^.

In this work we investigate how the wiggle and ripple structures are affected by a transition into a non-spherical shape. We further briefly discuss how an absorptive sample and the numerical aperture affect the wiggles and ripples (Details can be found in the Supplementary Materials).

In order to investigate the effect of shapes different from spheres and the validity of the van de Hulst formula, we investigate a model system that allows to perform the transition from a sphere to a system that shows irregular scattering features. For this purpose, we study the transition from a circular scatterer to a stadium-shaped scatterer. The stadium-shaped scatterer is inspired by the Bunimovich stadium, which is an irregular, chaotic system^34,35^. The transition from a regular to a chaotic system can, e.g., be studied by introducing a parameter that changes the geometry of the system such that the parameter for a certain range refers to a non-chaotic system and for another range to a chaotic system. In our case this means that when changing the parameter *d* of the billiard (see Fig. 1d), the system can change from a regular system for $d=0$ (the disk), to a chaotic system for $d>0$.We were especially interested in how the transition from a regular to a chaotic system affects the extinction efficiency, $Q_{ext}$, and the apparent absorbance spectra. In this work we investigate this model system by (1) deriving an approximation formula for the extinction efficiency for the stadium-shaped scatterer following the van de Hulst approach^20^, (2) evaluating numerically exact electromagnetic simulations, and (3) classical ray tracing. For calculating the electromagnetic near- and far-field of arbitrary-shaped scatterers, the wave optics module of the COMSOL Multiphysics software was used^42^.

### Approaches for ray tracing and assessment of classical chaotic scattering

In order to determine if the system is chaotic or not, we study the behavior of classical rays. Chaotic scattering systems have been studied for quantum wave systems and the corresponding classical ray systems^33,35^. Using the theory from this field, we evaluate if the system is chaotic or regular. The shape of the scatterer in this study is inspired by the *Bunimovich stadium*, which is a chaotic system. In contrast to the scattering situation that is considered in our study, the Bunimovich stadium has been originally studied as a closed system, where the rays cannot leave the system^34^. A chaotic scattering system is extremely sensitive to initial conditions. In a regular scattering system, a slight change in initial conditions of the rays, i.e., their starting positions, will be followed by only small differences in the paths of the corresponding rays. In the case of a chaotic system, small changes in starting conditions will be followed by large differences in the paths of the corresponding rays.

In our case of an open scattering system, rays are sent in from the top of the scatterer and are refracted into the system. The rays are treated as Newtonian rays, so when a ray hits a boundary it will either be transmitted or stay in the system due to total internal reflection. We can qualitatively show the occurrence of chaos by evaluating the lengths of the rays inside the scatterer as a function of initial position. The result is a path-length plot^30,31,33,35,43^, an established tool in chaotic scattering. It can be used as a diagnostic tool for the presence or absence of chaos and can be used to distinguish chaotic systems, exhibiting chaotic scattering, from regular systems that are not chaotic and show no chaotic scattering. For instance, a path-length plot may reveal the presence of extremely long paths, a necessary condition for chaotic scattering^30,31,33,35,43^. In addition, when the path lengths are rapidly changing for a slight change in initial conditions, this is a further indication of chaos. For a sphere, for instance, i.e., a regular system without chaotic scattering, both long-lived rays, i.e., long path lengths, and sensitivity, are absent. We see this in the following way. Since a sphere is composed of an optically denser material than the surrounding atmosphere, total internal reflection cannot occur when a ray that has entered a sphere impinges again on the surface of the sphere from the inside. Therefore, long-lived Newtonian rays do not exist when the scatterer has a spherical shape. However, by transforming the circular scatterer into a stadium-shaped scatterer, long-lived Newtonian rays can occur due to total internal reflection rays that are guided out of the semi-spherical end caps and into the straight “channel” section of the stadium.

In this work we evaluate the behavior of the classical rays for a system where the ripples have disappeared, i.e. we do not observe the standing waves in the scatterer. This system corresponds to systems without ripples in the absorbance spectra that we observe in FTIR experiments.

Several factors may be considered in order to decide whether the scattering behavior of the system is chaotic. In this work we consider (1) the fractal structure of path-length plots, (2) their fractal dimensions, and (3) the Lyapunov exponent of the system.

By magnifying a path-length plot several times, fractal structure may emerge. A fractal is a class of complex structures that shows self-similarity or approximate self-similarity on all scales^44^. Typically, a fractal is a point set embedded in *D* dimensions, and therefore, at first sight, appears to be an object of dimension $D-1$. A celebrated example is the coastline of Britain^44^. One might think that the coastline of Britain is a one-dimensional object, being, well, a line, but it turns out that this line is so complex, that in some ways it resembles a two-dimensional object. The “compromise” is to associate a fractal dimension, i.e., a non-integer dimension with the coastline of Britain, a dimension that is between 1 and 2. Thus, the fractal dimension characterizes the complexity of a fractal. The mathematics of how this is done is exact^31,32,44^. For instance, as explained below, we will use the method of box counting^31^ in our detailed analysis of path-length fractals. Apart from Britain’s coastline, many other objects in nature have a fractal structure. Examples are cumulus clouds^45^, trees^46^, and lungs^47^. A model fractal, for its simple rule of construction extensively studied in mathematics and biology, is the Cantor set^47^. Depending on the type of scatterer, our path-length plots may also have a fractal structure. If fractal structure is present, and if the starting position of a ray coincides with the fractal, a slight change of the ray’s starting position results in a considerable change of its path length. Thus, presence of a fractal indicates the presence of chaos.

In order to evaluate the fractal dimension of path-length plots, we apply the box-counting method^31^. By dividing the set of rays into smaller and smaller subsets, and counting the number of sets that contain long-lived rays, the fractal dimension can be obtained as the slope of a logarithmic plot of the number of subsets with at least one long-lived trajectory and minus the width of the subset^48^. A non-integer (fractal) dimension is an indication of chaos^30–32,49^.

The Lyapunov exponent can be found by evaluating the distance between two neighboring long-lived rays as a function of time^30–32,49^. A positive Lyapunov exponent is an indication of chaos^30–32,49^. Thus, fractal dimension and Lyapunov exponent are two complementary indicators and measures of chaos. While the fractal dimension is a static, global measure, that deals with the entire set of scattering rays at once, the Lyapunov exponent is a dynamic, more local measure that characterizes the dynamical properties of selected rays. For the interested reader we refer, e.g., to^30,50^ for a further introduction to fractals, Lyapunov exponents, and chaotic scattering.

In order to support the relevance of the theory of the systems investigated here to the field of infrared spectroscopy, we present and evaluate three measured absorbance spectra. The measured samples are chosen to have spherical, quasi-spherical, and non-spherical shape, i.e., PMMA spheres, pollen grains, and human lung cancer cells, respectively. The spectrum of the PMMA (polymethylmethacrylate) sphere was recorded at the SMIS infrared beamline at the SOLEIL synchrotron in France. Microspheres of PMMA with a diameter of approximately $15\,{\upmu \rm m}$, deposited on ZnSe slides, were measured in transmission mode. A Nicolet 5700 FTIR spectrometer with a Nicolet Continuum XL IR microscope, coupled with the synchrotron infrared beam, was used for the measurements. The Juniperus Excelsa pollen samples, representing a quasi-spherical biological system, were collected at the Botanical Garden of the Faculty of Science of the University of Zagreb in 2012, and were measured at the same infrared beamline, with the same instrumental setup. The diameter of the pollen grains was estimated to be between 10 and $40\, {\upmu \rm m}$. More detailed information about measurement and analysis of the PMMA and pollen spectra can be found in reference^22^. Finally, a human lung cancer cell, represents an example of a non-spherical biological sample. The spectrum was recorded at the European Synchrotron Radiation Facility (ESRF) in Grenoble, at the ID 21 beamline. For these measurements, a Nicolet Nexus 870 spectrometer coupled with a Continuum-Thermo Nicolet microscope was used. More details of the experimental setup can be found in^10^. The cells used in this experiment were obtained from the non-small cell lung cancer (NSCLC) cell line SK-MES purchased from the European Collection of Cell Cultures (Salisbury, U.K.)^10^.

In our numerical studies and simulations, we consider scatterers with a constant refractive index. In the case of a cell, this may be interpreted as the effective index of refraction of the entire cell.

## Results

**Figure 2:**
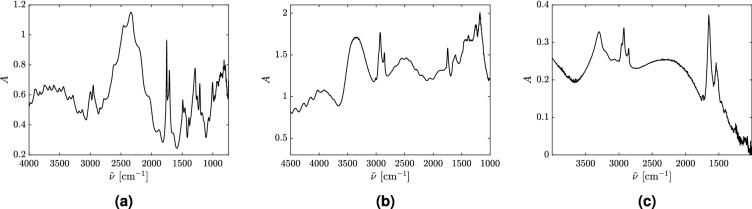
Absorbance spectra of (**a**) a PMMA-sphere^22^ (**b**) a Juniperus Excelsa pollen grain^22^ and (**c**) a human lung cancer cell^10^. In order not to introduce spurious features into the spectra that may result from using a correction algorithm, and in order to highlight the original appearance of the spectra, all three spectra shown are uncorrected, raw spectra. The tiny, sharp features seen in the spectrum in frame (**c**) are mostly due to noise and counting statistics; they are not reproducible ripples.

In order to corroborate the theoretical results presented in this paper, FTIR raw spectra of PMMA-spheres, pollen, and human cells were measured and analyzed. Three of the spectra are shown in Fig. 2.

The absorbance spectrum for a PMMA-sphere (Fig. 2a) shows both ripples and wiggles, consistent with Mie theory. The spectrum of the Juniperus Excelsa pollen grain (Fig. 2b) also shows both wiggles and ripples. This is expected since the shape of the pollen grains, whose spectrum is shown in Fig. 2b, is close to spherical. In the case where the scatterer has a non-spherical shape, e.g., a biological cell (Fig. 2c), the corresponding spectrum only shows the wiggles, i.e., the broad oscillations, but not the ripples.

### Developement of an approximation for $Q_{ext}$ for stadium-shaped scatterers

As presented above, the van de Hulst approximation for $Q_{ext}$ for a sphere is used in the ME-EMSC algorithm. In order to derive an analytical van-de-Hulst-type extinction formula for our stadium system, we start by looking at an approximation for a disk and follow the same procedure as presented by van de Hulst^20^ for a sphere.

By following the same procedure as presented by van de Hulst^20^, we found an approximation formula for the extinction efficiency of a disk according to

3 $$\begin{aligned} Q_{ext}(\rho ) = 2 - 2 J_0(\rho ) + 4 \sum _{n=1}^{\infty } J_{2n}(\rho ) \frac{1}{4n^2-1}, \end{aligned}$$

where $J_0$ is the 0th order Bessel function of the first kind and $J_{2n}$ are Bessel functions of the first kind and order 2*n*. $\rho =2ka(m-1)$, where *k* is the angular wavenumber, *a* is the radius of the disk (as shown in Fig. 1d), and *m* is the refractive index of the scatterer. Details are given in Supplementary Materials Sec. C.

**Figure 3:**
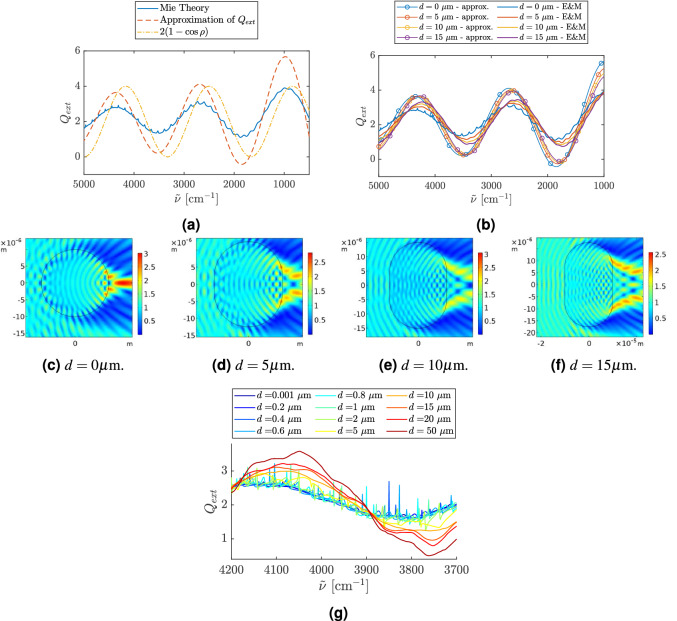
In frame (**a**), the exact extinction efficiency is shown for a cylinder in the case where the *E*-field is parallel to the cylinder axis (blue line). The radius of the cylinder is 10 $\upmu$m and the refractive index is 1.3. The red dashed line shows the approximation of $Q_{ext}$ given in Eq. (3). The yellow line is the extinction efficiency the stadium shaped scatterer will approach according to Eq. (4) when $d>>a$ in the case where the thickness of the straight part is 20 $\upmu \rm m$ and the refractive index of the scatterer is 1.3. Frame (**b**) shows $Q_{ext}$ of a stadium with a refractive index of 1.3 and a radius of the circular end caps equal to 10 $\upmu \rm m$. The length of the straight sections is 0 $\upmu$m (blue lines), 5 $\upmu$m (red lines), 10 $\upmu$m (yellow lines) and 15 $\upmu$m (purple lines). The figure shows that the wiggles are shifted to the right both according to the COMSOL Multiphysics simulations of the electromagnetic field (solid thick lines) and according to the approximation of $Q_{ext}$ (thin lines with circles) given by Eq. (4). For the frames (**c**)–(**f**), the radius of the circular end caps is 10 ${\upmu \rm m}$ and the refractive index of the scatterer is 1.3. Frames (**c**)–(**f**) show the norm of the E-field in the case where a plane wave is incident from the left with wavenumber ${\tilde{\nu }} = 2600$ $\mathrm {cm}^{-1}$. Frame (**g**) shows $Q_{ext}$ as a function of wavenumber. The change in $Q_{ext}$ is illustrated as a function of the length *d* of the straight sections of the stadium as we change *d* from 0.001 (dark blue), which is close to a disk, to a stadium with $d=50\,\upmu$m (dark red). The radius of each of the endcaps is 10 $\upmu$m and the refractive index of the stadium is 1.8. The simulations are done by COMSOL Multiphysics.

Figure 3a shows the result of Eq. (3) together with the exact Mie solutions for an infinite cylinder. As for the sphere in Fig. 1b, the approximation predicts the positions of the wiggles, but not the ripples. The graph of the approximation formula of Eq. (3) is scaled with respect to the exact result. This is not a problem for state of the art Mie scatter corrections in infrared spectroscopy, since the Mie scatter correction is scaling invariant with respect to the theoretical model used for the correction^10,12,14,16^.

An approximation can also be found for a stadium shaped scatterer (details are given in Supplementary Materials Sec. C). We arrive at the following explicit formula for the extinction efficiency

4 $$\begin{aligned} Q_{ext}(\rho ) = \frac{2a}{2a+d} \left( 2 - 2 J_{0}(\rho ) + 4 \sum _{n=1}^{\infty } J_{2n}(\rho ) \frac{1}{4n^2-1} \right) + \frac{2d}{2a+d} \left( 1 - \cos {(\rho )} \right) , \end{aligned}$$

where *a* and *d* are the radius of the stadium’s end caps and the lengths of the straight sections as indicated in Fig. 1d, and $\rho$ is the same as described above.

In Fig. 3b, we compare the results of Eq. (4) with electromagnetic COMSOL Multiphysics simulations. The figure shows that both the van de Hulst formula in Eq. (4) and the COMSOL Multiphysics simulations predict that for a stadium, the positions of the wiggles are shifted to the right as *d* increases. This is explained in the following way. According to Eq. (4), the Bessel term dominates for small *d*, while for large *d* the cosine term dominates. The Bessel term, i.e., $Q_{ext}$ for a sphere, is illustrated as the red, dashed line in Fig. 3a, while the the function $2(1-\cos (\rho ))$, essentially the cosine term in Eq. (4), is illustrated as the yellow, dash-dotted line in Fig. 3a. Fig. 3a shows that the cosine term is shifted to the right with respect to the Bessel term. Thus, because of the switch-over from the Bessel term to the cosine term in Eq. (4) as a function of increasing *d*, a right-shift occurs in $Q_{ ext}$ as a function of increasing *d*, as observed in Fig. 3b.

### Investigation of the electromagnetic field for stadium-shaped scatterers

To evaluate the scattering properties of a stadium further, the extinction coefficient and the electric field were calculated using COMSOL Multiphysics^42^. Figure 3b shows the results of these simulations. The refractive index was set to 1.3 and the radius of the half disks at the end of the stadium was set to 10 µm.

Figure 3b shows the extinction efficiency $Q_{ext}$ as a function of the wavenumber found by COMSOL Multiphysics (thick lines) together with the approximation for $Q_{ext}$ according to Eq. (4) (thin lines with circles), where the blue curves show the respective extinction efficiencies for a disk. In the exact result for a disk (blue, thick line in Fig. 3b), the familiar patterns of wiggles and ripples are present. As we increase *d*, the sharp ripples disappear. However, we still observe some remnants of ripples at the smallest wavenumbers.

In Fig. 3c–f, the norm of the $\vec{E}$-field is shown for wavenumber ${{\tilde{\nu }}} = 2600$ $\mathrm {cm}^{-1}$. The plane wave is propagating towards the scatterer from left. In the case of a circular scatterer (Fig. 3c), we clearly see the focusing effect and the photonic jet behind the scatterer. As *d* increases, we observe that the photonic jet behind the scatterer divides into two. These two jets emerge from the scatterer at the points where the straight sections of the stadium meet the circular end caps of the stadium. This observation is well-known from the field of micro-disk lasers^51^.

In order to further evaluate how the transition to chaos affects the system, the refractive index of the scatterer was increased to 1.8. Figure 3g shows $Q_{ext}$ for this case, which was found from simulations of the electromagnetic field (by COMSOL Multiphysics). The wavenumber interval is between 4200 and 3700 $\hbox {cm}^{-1}$. The colors indicate the size of *d*, which ranges from 0.001 µm (dark blue line) to 50 µm (dark red line). We notice that compared to Fig. 3b the resonances (ripples) in Fig. 3g are much more pronounced. This is explained in the following way. An increased ratio of the refractive index between the disk/stadium and the surroundings (1.8 in Fig. 3g compared with 1.3 in Fig. 3b) results in an increased probability of total internal reflection, which, in turn, results in sharper ripples.

For the cases where *d* is equal to 2 µm or less, the ripple structure is present. Investigating the near field at wavenumbers that correspond to sharp peaks in $Q_{ext}({\tilde{\nu }})$, we observe that they correspond to standing waves. Supplementary Materials Sec. D shows plots of the electric field. On the way to a more deformed disk, we observe that for a weakly deformed disk the circular standing-wave pattern transforms into a diamond-shaped pattern. The diamond orbit in the Bunimovich billiard has a total of four bouncing points: two in the middle of the perimeter of the half spheres on both ends of the billiard and two in the middle of the rectangular part of the billiard (upper and lower sides). It has therefore the shape of a diamond^52^, which explains its name. At $d=5$ µm, nearly all the ripples have disappeared. Some small peaks are still observed.

As Fig. 3g indicates, the sharp ripples disappear when *d* is 10 µm and larger. For the cases where the sharp resonances have disappeared, we observe scarlets^53,54^ (see Supplementary Materials Sec. D), which is an indication of chaos. The scarlets are rigde-structures that show how the waves are locally guided.

The ratio between the wavelength and the size of the scatterer is important in order to observe wave chaos. It is therefore possible to observe classical chaos in cases where we still have regular scattering in the wave picture. On the other hand, in cases where $d = 10$ µm and larger, the ratio between the wavelength and the size of the scatterer is so small that the simulations of the electromagnetic field indicate chaotic behavior as well. Thus, in the latter case, both the classical and electromagnetic simulations agree.

### Behavior of classical rays for a stadium-shaped scatterer

In order to evaluate if the system is chaotic or not, a ray tracing program was written. The program simulates the evolution of rays sent straight down toward the scatterer as shown in Fig. 1d. In order to look for an indication of chaos, the path lengths of the rays are evaluated for systems with different lengths of the straight sections *d* and different refractive indices. Only rays sent towards the left half disk of the stadium are considered. The reason is that (i) rays incident on the straight sections of the stadium pass straight through and are therefore uninteresting in the context of chaos, and (ii) because of symmetry, rays incident on the right end cap behave exactly the same as rays incident on the left end cap.

**Figure 4:**
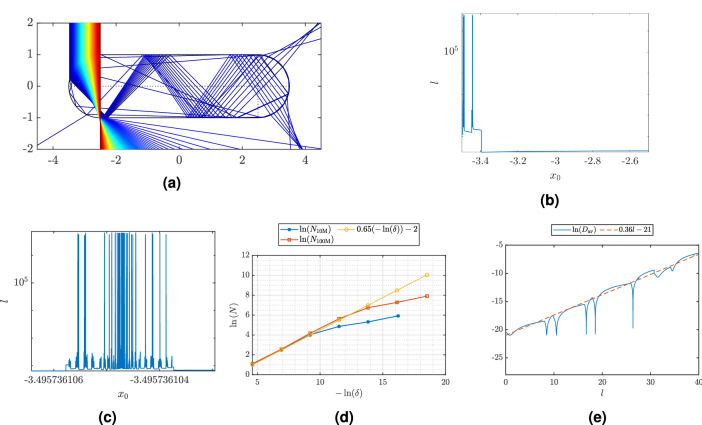
Frame (**a**) shows how rays sent straight downwards toward the left end cap refract when hitting the scatterer. Frame (**b**) indicates the length of the each ray, *l*, inside the scatterer as a function of start position $x_0$ with a logarithmic *y*-axis. Frame (**c**) also indicates the length of each ray as a function of start position. A selected interval within the $x_0$-interval in frame (**b**) has been magnified, a new $x_0$-interval is selected and magnified and so on. In frame (**c**), the $x_0$-interval has been magnified 7 times. The fractal structure is preserved. (See Supplementary Materials Sec. E for further magnifications revealing the fractal structure.) Frame (**d**) shows the determination of the fractal dimension of the set of long-lived rays based on the box-counting method. The blue and red lines show $\ln (N)$ as a function of $-\ln (\delta )$ where *N* is the number of intervals that contain at least one long-lived trajectory and $\delta$ is the width of the interval. The slope of the blue and red lines are indicating the fractal dimension. The yellow line indicates that the slope is 0.65. $N_{\mathrm{{10M}}}$, the blue line, indicates *N* for the case where 10 million rays were started in the interval and $N_{\mathrm{{100M}}}$, the red line, where 100 million rays were started in the same interval. Frame (**e**) shows the average of $\ln (D)$, where *D* is the distance between the two rays, as a function of path length, *l*, for 10 pairs of rays. The red, dashed line indicates the slope, i.e. the Lyapunov exponent, which in this case is 0.36. For all the frames, the refractive index of the scatterer is 1.8. Classical ray tracing investigations are wavelength independent and the length of the straight part of the stadium is 5 times the radius of the end caps. This corresponds to a system where $d=50 \, {\upmu \rm m}$ and $r=10\,\upmu \rm m$.

In Fig. 3g we observe that in the case where the length of the straight sections of the stadium, *d*, is 5 times larger than the radius, *a* (see Fig. 1d), the ripples disappear and we observe scarlets in the corresponding electric-field plots. In this case, the refractive index of the scatterer is 1.8. Since classical rays are independent of wavelength, *a* was set to 1 and *d* was set to 5. Figure 4a shows the resulting behavior of the simulated rays and Fig. 4b shows the lengths of the rays inside the scatterer as a function of starting position. For rays incident on the very left of the stadium, long-lived trajectories are observed. This is an indication of chaos.

In order to investigate whether the life-time plot of the rays exhibits fractal structure, one tenth of the range of starting positions in Fig. 4b was magnified. This was repeated seven times. The last of these path length plots is shown in Fig. 4c. Fractal structures are present in all seven stages of magnification, all plots and details are shown in Supplementary Materials Sec. E.

The fractal dimension of the system was found by evaluating a system where 10 million rays were sent straight downwards and incident on the left end-cap. A long-lived ray was defined as a ray with a length longer that 1 000 000. This set of starting positions was divided into subsets of length equal to $\delta$. The blue line in Fig. 4d shows a plot of $\ln (N)$ as a function of $-\ln (\delta )$, where *N* is the number of subsets that contain at least one long-lived ray at resolution $\delta$. The fractal dimension is then found as the slope of $\ln (N)$ as a function of $-\ln (\delta )$.

Figure. 4d shows $\ln (N)$ in the case where in total 10 million (blue line) and 100 million (red line) rays are started in the interval from $x_0 = -3.5$ and $x_0 = -3.4$. The yellow line is a straight-line fit to the data and shows that the slope, i.e., the fractal dimension, is approximately 0.65. This can be compared with the fractal dimension $\ln (2)/\ln (3)=0.63$ of the Cantor fractal^31,47^. Incidentally this is also the same as the fractal dimension of a certain repellor set in the 3-disk scattering system^55^ that has been proposed as a model for unimolecular fragmentation. Thus, our value of 0.65 for the fractal dimension of the path-length plot is very similar to the fractal dimension of the paradigm Cantor fractal and a fractal that occurs in the 3-disk scattering system that shows fully developed chaotic scattering^55^. Since the value of 0.65 found for our path-length fractal is close to midway between 0 and 1, this also shows that, like in the case of the Cantor and 3-disk fractals, chaotic scattering is important in our system. Had the fractal dimension been closer to 0, this would have indicated a relatively minor role of chaotic scattering in our system.

As the figure shows, $\ln (N)$ deviates from a straight line when $\delta$ becomes too small. This is due to the fact that the resolution of the life-times (i.e., 10 million rays or 100 million rays) is still not enough for the corresponding $\delta$ where the bending-over of $\ln (N)$ versus $-\ln (\delta )$ occurs.

In order to evaluate the Lyapunov exponent we evaluated the average of the Lyapunov exponent for 10 rays. This is shown in Fig. 4e. In Supplementary Materials Sec. F, plots of each of the 10 rays and information about their start position are given. The dips in the blue line in Fig 4e arise from the fact that the rays are crossing each other. The slope of the blue line in Fig 4e (ignoring the dips) is the Lyapunov exponent. The red, dashed line indicates that the slope is approximately 0.36. This can be compared with the Lyapunov exponent computed by Benettin and Strelcyn of $\approx 0.4$^56^ for the confining Bunimovich stadium billiard with the same ratio of end-cap radius and length of straight section as in our example. Our Lyapunov exponent for the scattering stadium billiard is also of the same order as the Lyapunov exponent for a Sinai billiard^31,32^ of approximately the same size, namely $\approx 2\ln (2)\approx 1.4$^57^. It is not surprising that the Sinai-billiard value is about a factor 4 larger since the Sinai billiard in some sense is more “compact” and lacks the long straight section of the Bunimovich billiard that does not contribute to the exponential divergence of rays. We may also compare our value of 0.36 to the Lyapunov exponent of the logistic map^31^, a dynamical system extensively used in biology and population dynamics. At the map control parameter $r=4$, the logistic map is fully chaotic and has a Lyapunov exponent of $\ln (2)\approx 0.7$^31^. These examples show that the Lyapunov exponent of 0.36 found in our stadium scattering system is in the same ball park as the Lyapunov exponents in similar chaotic dynamical systems. This also shows that chaotic scattering of the Bunimovich billiard is significant enough to appreciably influence its scattering properties, as evidenced, e.g., by the reduction, and even absence of sharp scattering features (ripples). Thus, understanding the underlying chaoticity of the scattering process profoundly benefits our understanding and characterization of scattering effects in complex samples.

### Elliptic deformation

**Figure 5:**
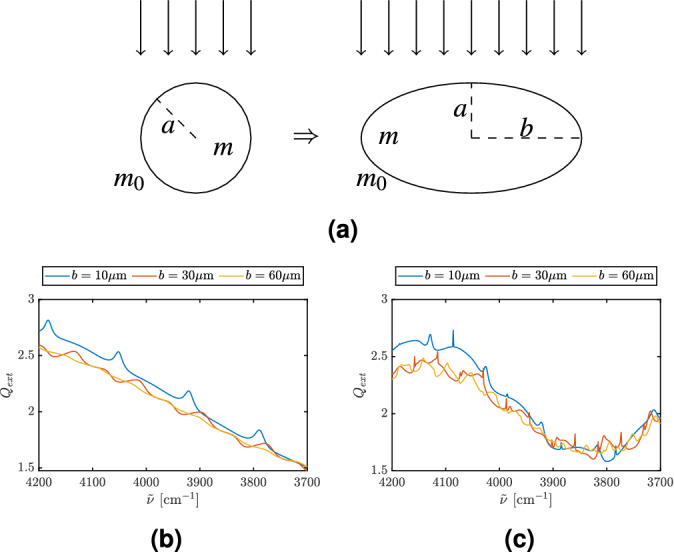
We are evaluating a system that transforms from a disk with radius *a* into an ellipse. The parameter that describes the height of the ellipse is its semi-major axis, *a*, which is kept constant and equal to the radius of the disk. We deform the scatterer by increasing the semi-major axis, *b*, of the ellipse. The refractive index of the scatterer is *m* and the refractive index of the surroundings is $m_0 = 1$. The light is entering the system from above. Frame (**b**) and (**c**) show how $Q_{ext}$ changes as the deformation of the ellipse increases. The semi-major axis, *a*, of the ellipse [see frame (**a**)] is kept constant at $a=10\,\upmu$m, and *b* is selected to be 10 µm (blue line, i.e., a disk-shaped scatterer), 30 µm (red line) and 60 µm (yellow line). The refractive index is (**b**) 1.3 and (**c**) 1.8.

The following question arises: Is the disappearance of the ripples of the system *caused* by the chaotic behavior or is the reason for the disappearance of the ripples primarily caused by the deformation into a stadium, and the chaotic scattering is merely a by-product of deformation, i.e., the disappearance of the ripples is *correlated* with the onset of chaotic scattering, but not *caused* by chaotic scattering. In order to conclusively answer this question, we also considered the deformation of the disk into an ellipse, which is an integrable system that has no chaotic scattering at any deformation. Therefore, if the ripples disappear under deformation into an ellipse, we have conclusively shown that the cause for the disappearance of the ripples is deformation, and that chaotic scattering, a consequence of deformation, is merely correlated with the disappearance of the ripples, but not the cause. To investigate this point, we studied how the ripple structure is changing as we deform our disk-shaped scatterer into an elliptical scatterer as shown in Fig. 5a.

An ellipse is described by the two semi-major axes *a* and *b* as shown in Fig. 5a. In our deformation studies, we increase the width of the ellipse in *x*-direction, i.e., we increase the semi-major axis *b*; the incident light illuminates the scatterer from above. The semi-major axis, *a*, is selected to be 10 µm, and *b* is increased from 10 µm (i.e. a disk-shaped scatterer) to 60 µm. Figure 5b,c show how the extinction efficiency changes as we increase *b*. We observe that the ripple structure disappears when *b* becomes large enough. However, we also see that compared to the stadium, the ripples are more resilient in the case of the ellipse, i.e., an integrable system, and disappear only at a much larger deformation compared to the stadium, which exhibits chaotic scattering. This shows conclusively that the deformation is the reason for the disappearance of the ripples. However, we also see that chaotic scattering has an accelerating effect on the disappearance of the ripples, i.e., the ripples disappear more quickly in the presence of chaotic scattering. The calculations were done with COMSOL Multiphysics^42^. Contrary to the sensitivity of the ripples we observe that the wiggle structure is robust with respect to deformation. This is analogous to our observations in the case of the stadium-shaped scatterer (Fig 3b).

In Supplementary Materials Sec. G, the electric field is shown for selected wavenumbers for the systems evaluated in Fig. 5. The selected wavenumbers correspond to peaks (ripples) in the $Q_{ext}$.

## Discussion

In order to study the influence of a scatterer’s shape on FTIR spectra, we chose to compare the scattering properties of elliptical shapes and stadium billiards. The choice of those systems is natural, since they allow us to study the transition from an integrable scattering system to a chaotic scattering system, whose properties have to be investigated numerically. The commercially available software package Wave Optics for COMSOL Multiphysics^42^ allows us to extend previous knowledge accumulated in the domain of regular Mie scattering into the domains of regular scattering on an elliptic cylinder and chaotic scattering on a stadium-shaped cylinder. In both systems the smooth transition from the disk system to the ellipse and stadium systems allows us to follow the evolution of the known Mie features, such as wiggles and ripples, into the domains of deformed, regular scattering in the case of elliptic deformations, and chaotic scattering in the case of stadium-shaped deformations. In both cases we also looked at the dependence on the index of refraction. As one of our main observations we found that the ripples present in the scattering from a disk are suppressed by deformation, and faster in the presence of chaotic scattering (see Figs. 3b,g), compared to the case of regular scattering (see Figs. 5b,c).

Despite the fact that the real world is three-dimensional, in order to keep our systems simple, allowing us to focus on the essential mechanisms without cumbersome numerical ballast, we focused in this study on effectively two-dimensional (cylindrical) systems. This is justified since, as shown in Fig. 1b for a circular scatterer, qualitatively, and to some approximation even quantitatively, the same phenomena can be observed in two- and three-dimensional systems. Furthermore, a two-dimensional system is equivalent to a cylindrical three-dimensional system, i.e., a system which is translationally invariant in the third dimension.

Figure 3a shows how the approximation of the extinction efficiency for a disk, derived in Eq. (3), compares with the exact Mie solution of the system. In the case of a disk, our approximation of the extinction efficiency reproduces the positions of the wiggles, but lacks the ripples. When we use this approximation, we need to have in mind that the amplitudes of the resulting, approximate wiggles are too large. As the length of the straight sections of the stadium, *d*, increases, the COMSOL Multiphysics calculations show (see Fig. 3b) that the positions of the wiggles are shifted towards the right. The same shift to the right is observed if we evaluate the extinction efficiency using our approximation of the extinction efficiency according to Eq. (4). This is also illustrated in Fig. 3b.

We also observed some interesting structures in the electric field itself. A plot of the norm of the electric field shows how the photonic jet splits into two as the length of the straight sections of the stadium increases (see Fig. 3c–f and Supplementary Materials Sec. D). The two jets emanate from points in the circumference of the stadium where the end caps merge with the straight sections. Similar effects have first been observed in the field of micro-disk lasers^51^ and are used technically for coupling radiation in and out of these lasers. We also saw whispering gallery modes of higher than first order forming concentric rings in the $\vec{E}$-field patterns located close to the perimeter. Further, we also saw the manifestation of a “scar”^58^, i.e., the local enhancement of the electric field in the vicinity of the diamond orbit of the Bunimovich stadium. Lastly, we also observed the phenomenon of “scarlets”^53,54^ in the region of deeply chaotic scattering.

In order to further evaluate the stadium-shaped system, we studied the behavior of the classical rays in the system. For the system where we observe scarlets in the electric field, we also observe that the length of the rays is very sensitive to their initial positions, which is an indication of chaos. In order to further evaluate the classical scattering properties of the system, we found indications of chaos in terms of (i) the emergence of a fractal structure, which we traced through seven generations of magnification (see Fig. 4c and Supplementary Materials Sec. E), (ii) a non-integer fractal dimension (see Fig. 4d), and (iii) a positive Lyapunov exponent (see Fig. 4e and Supplementary Materials Sec. F). We observe that the fractal structure of the system is not self-similar. This contrasts with the self-similar structure found in^35^. However, we were able to reproduce the self-similar fractal structure reported in^35^ when we used precisely the same conditions and parameters as in^35^. So, we are sure that our numerical codes are correct and that the differences observed between our fractal structures and the fractal structures reported in^35^ are a consequence of vertical versus horizontal incidence of rays and a difference in the respective refractive indexes used by us versus the ones used in^35^.

Deformation is a new mechanism of ripple suppression that is now added to the mechanism of ripple suppression in the presence of damping^23^. We found that ripples are suppressed for deformations that lead to either integrable or non-integrable, chaotic systems. We also found that in integrable systems the ripples are more resilient to deformation compared with systems that exhibit chaotic scattering. Intuitively it is clear why the ripples are suppressed more efficiently in the chaotic scattering situation. As shown in Supplementary Materials Sec. D, whispering gallery modes, the origins of ripples, are not well, if at all, supported in the chaotic scattering situation, accounting for their fast disappearance.

Other mechanisms that could affect the ripples and wiggles are, e.g., the presence of absorption, the numerical aperture, and the rotation of the sample with respect to the direction of the incident beam. For instance, it has been shown (see, e.g.,^23^) that the sharp ripples, i.e., the needle-shaped resonances in the spectrum, start to disappear as soon as we turn on absorption. The effects of absorption and apertures can be studied using Mie Theory. This is done in Supplementary Materials (Sec. H and Sec. I), where we show that the broader ripples, which correspond to higher-order Mie resonances^23^, are still present until $A = 0.5$ is reached. Further, we observe that the ripples are present even if we take the numerical aperture into account. We observe that the curve of $Q_{ext}$ is shifted downwards as NA increases, which is also shown by^59^, but the ripple structure is preserved. Due to this fact, we would expect to observe the broader-type ripples in the non-absorptive regions in FTIR measurements. This is indeed confirmed by Fig. 2a,b, which are the spectra of spherical and quasi-spherical systems. However, the absorbance spectrum of the biological cell, shown in Fig. 2c, does not show a ripple-structure in the non-absorptive regions. This supports our hypothesis that the non-spherical shape of this scatterer results in a suppression of ripples combined with, possibly, chaotic scattering behavior.

We did not investigate the effect of rotation of the sample, but offer the following conjectures to be investigated in further studies. The rotation, i.e., the orientation, of the sample does not change the nature of the scatterer, i.e., regular (integrable) or chaotic, since regularity (integrability) and chaoticity are intrinsic properties of the scatterer, independent of sample orientation. Therefore, since our simulations indicate that there is a profound difference in the manifestation of ripples between regularly and chaotically scattering samples, and since the rotation angle does not change the nature of the sample, i.e., regular or chaotic, we conjecture that the orientation of the sample does not have a qualitative influence on the appearance or disappearance of the ripples, but only changes details, such as, possibly, their heights. This is corroborated by comparing our classical calculations on the stadium-shaped billiard with the billiard computations in^35^. The two cases differ in the direction of the incident beam, which corresponds to a sample rotation of 90 degrees. Yet, both computations yield very similar, although, as expected, not identical, scattering fractals, that, in an actual experiment on a stadium-shaped cylindrical sample would lead to similar appearances and disappearances of ripples in the corresponding sample-deformation regimes.

The results of this study also supports the ME-EMSC method of analysis, which uses the van de Hulst approximation in order to Mie-correct FTIR spectra. Although ME-EMSC is based on the Mie-wiggle structure and does not take the Mie ripples into account, neglecting the ripple structure is justified in most cases of biological significance, since the shape of most biological samples is non-spherical, resulting in an absence of ripples. This follows from the fact that integrable deformations, which would support the manifestation of ripples even for relatively large deformations (see Fig. 5) are rare oddities in the biological world, while generic deformations, more common in biology, result in chaotic scatterers with high probability^31,32,49^, and consequently fast destruction of ripples for relatively small deformations (see Fig. 3b,g). The absence of ripples in most biological systems is further supported by the experimental results shown in Fig. 2, where we see clearly how the ripples are present in measurements of perfectly spherical scatterers (PMMA-spheres) and nearly spherical scatteres (pollen). In the case of a non-spherical scatterer (biological cell) we observed that the ripples completely disappeared, even in non-absorptive spectral regions. Still, it is important to be aware of the occurrence of ripples in the case of near-spherical samples, which definitely also occur in FTIR spectroscopic applications, as shown, e.g., in Figs. 2a,b.

## Conclusion

In this paper, studying scattering on a disk that can be smoothly transformed into either an integrable elliptic scatterer or a chaotic Bunimovich stadium scatterer, we showed that deformation of the scatterer has a profound influence on the extinction efficiency. While regular scattering on a disk is accompanied by sharp resonances, i.e., whispering gallery modes that manifest themselves as “ripples” in the extinction efficiency that are supported up to relatively large deformations, the ripples are nearly completely destroyed, even for relatively modest deformations, in the presence of chaotic scattering. This is an important observation, since ripples may change both the locations and amplitudes of chemical absorption bands. While it is known that ripples are eliminated from FTIR spectra by sufficiently large absorption^23^, the fast and efficient destruction of ripples by chaotic scattering is both a new result and of immense practical importance for spectroscopic applications such as FTIR spectroscopy of biological cells and tissues. While ripples are certainly eliminated in the presence of both chaotic scattering and sufficiently large absorption, we observed that even in the absence of absorption, for instance in chemically inert spectral regions, the presence of chaotic scattering caused by sample deformation, is sufficient to suppress Mie ripples in FTIR spectra. This validates current correction methods for infrared absorbance spectra, such as ME-EMSC, in most situations of practical spectroscopic interest, in particular as applied to the correction and analysis of irregularly-shaped biological samples. Still, as shown by our results, great care has to be exercised when investigating quasi-spherically or quasi-elliptically deformed samples which do not suppress Mie ripples, which then may interfere with chemical absorption bands and may cause large errors in the analysis of spectra if Mie ripples are not properly taken into account.

## Supplementary Information

Supplementary Information.
