# Supplementary material for: The effect of deformation of absorbing scatterers on Mie-type signatures in infrared microspectroscopy
Source: Sci Rep. 2021 Feb 25;11:4675. doi: 10.1038/s41598-021-84064-5 (PMC7907113; doi:10.1038/s41598-021-84064-5)
Supplement: Supplementary file 1 — Supplementary Information. [file 41598_2021_84064_MOESM1_ESM.pdf]

Supplementary Material

The effect of deformation of absorbing scatterers on Mie-type signatures in infrared microspectroscopy

Maren Anna Brandsrud[1,*], Reinhold Blümel[2], Johanne Heitmann Solheim[1], Achim Kohler[1]
[1] Faculty of Science and Technology, Norwegian University of Life Sciences, Ås, Norway
[2] Department of Physics, Wesleyan University, Middletown, Connecticut, United States of America
[*] maren.brandsrud@nmbu.no

A Mie solutions for a soft disk

The behavior of light which is propagating towards a sphere or a cylinder is exactly described by Mie Theory[1]. The quantity Q_{ext} is dimensionless and describes the amount of light removed from forward direction. Q_{ext} is related to the scattering efficiency Q_{sca} and the absorption efficiency Q_{abs} according to

$$Q_{ext} = Q_{sca} + Q_{abs}. \tag{A.1}$$

For perfectly spherical scatterers, Q_{ext}, Q_{abs} and Q_{sca} are described exactly by the Mie Theory[1,2].

In this work, we restrict ourselves to work with a two-dimensional system. In the case where the scatterer is circular, the system is equivalent to the case where the electric field is parallel to the cylinder axis. The respective extinction, scattering and absorption efficiencies are given by

$$Q_{ext} = \frac{2}{ka} \sum_{n=-\infty}^{\infty} \Re(b_n), \tag{A.2}$$

$$Q_{sca} = \frac{2}{ka} \sum_{n=-\infty}^{\infty} |b_n|^2, \tag{A.3}$$

and

$$Q_{abs} = Q_{ext} - Q_{sca}. \tag{A.4}$$

k is the angular wavenumber of the incoming plane wave and a is the radius of the cylinder. The coefficient b_n is given by

$$b_n = \frac{\tan\beta_n}{\tan\beta_n - i}, \tag{A.5}$$

and $\tan\beta_n$ is given by

$$\tan\beta_n = \frac{mJ_n'(mka)J_n(ka) - J_n(mka)J_n'(ka)}{mJ_n'(mka)N_n(ka) - J_n(mka)N_n'(ka)}. \tag{A.6}$$

m is the refractive index of the cylinder, J_n is the nth order Bessel function of the first kind and N_n is the nth order Bessel function of second kind (Neumann function). The derivation of the above equations can be found in e.g. *Light scattering by small particles*, van de Hulst 1981[1].

B Scattering from a soft disk

A plane wave with a wavelength equal to λ is propagating from the left towards a circular scatterer as shown in Fig. B.1. The wave function of the incoming wave is given by ψ_0,

$$\psi_0 = e^{ikx} = e^{ikr\cos\theta}, \tag{B.1}$$

where k is the angluar wavenumber of the incoming plane wave, given by $\frac{2\pi}{\lambda}$. The x-position can be re-written in polar coordinates. ψ_0 can be written in terms of Bessel functions

$$\psi_0 = \sum_{l=-\infty}^{\infty} i^l J_l(kr) e^{il\theta}, \tag{B.2}$$

where J_l is the Bessel function of the first kind and order l.

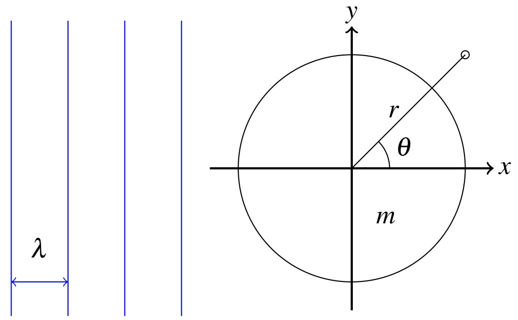

Figure B.1. The circular scatterer with refractive index m. A plane wave is incident from the left with a wavelength equal to λ.

The scattered wave, ψ_s, can be written in terms of outgoing Hankel functions

$$\psi_s(r,\theta) = \sum_{l=-\infty}^{\infty} A_l H_l^{(+)}(kr) e^{il\theta}, \tag{B.3}$$

where $H^{(+)}$ is the Hankel function of the first kind and order l, and A_l is the scattering amplitude. The wave function outside the scatterer is therefore given by

$$\psi_{out}(r,\theta) = \psi_0 + \psi_s = \sum_{l=-\infty}^{\infty} i^l J_l(kr) e^{il\theta} + \sum_{l=-\infty}^{\infty} A_l H_l^{(+)}(kr) e^{il\theta}, \quad \text{for } r > R\,. \tag{B.4}$$

The wavefunction inside the scatterer is given by ψ_{in} and can be written as

$$\psi_{in}(r,\theta) = \sum_{l=-\infty}^{\infty} B_l J_l(\kappa r) e^{il\theta}, \quad \text{for } r < R\,, \tag{B.5}$$

where κ is the angular wavenumber inside the scatterer, given by $\kappa = kn$, where n is the refractive index of the scatterer.

By requiring a continuous wave function and its derivative for ψ_{in} and ψ_{out} at the boundary of the scatterer, we can derive expressions for the coefficients A_l and B_l:

$$A_l = \frac{-i^l\left[\kappa J_l'(\kappa r) J_l(kr) - k J_l'(kr) J_l(\kappa r)\right]}{\kappa J_l'(\kappa r) H_l^{(+)}(kr) - k J_l'(\kappa r) H_l'^{(+)}(kr)}, \tag{B.6}$$

$$B_l = \frac{i^l\left[k H_l^{(+)}(kr) J_l'(kr) - k H_l'^{(+)} J_l(kr)\right]}{\kappa J_l'(\kappa r) H_l^{(+)}(kr) - k J_l(\kappa r) H_l'^{(+)}(kr)}. \tag{B.7}$$

C Development of an approximation for Q_{ext} for stadium-shaped scatterers

In order to derive an analytical van-de-Hulst-type extinction formula for our stadium system, we start by looking at an approximation for a disk and follow the same procedure as presented by van de Hulst for a sphere[1]. For a sphere, the extinction efficiency is given by[1]

$$Q_{ext} = \frac{4\pi}{k^2}\frac{1}{g}\Re[S(0)], \tag{C.1}$$

where k is the angular wavenumber, $g = \pi a^2$ is the geometrical cross section of the sphere (see Fig. 1a) and $S(0)$ is the amplitude function in forward direction.

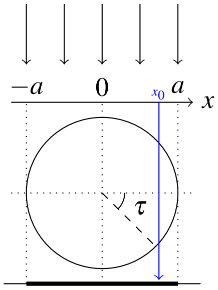

Figure C.1. System investigated for the evaluation of the extinction efficiency of a disk.

In order to derive the approximation for a circular disk, we evaluate how the rays, assumed to propagate straight through the scatterer, affect the wave in the geometrical shadow of the disk (the shaded area in Fig. C.1). The disk has a radius a and a refractive index m. The blue ray at $x = x_0$ has a phase lag equal to $2a\sin(\tau)(m-1)k$, where k is the angular wavenumber and $2a\sin(\tau)$ is the length of the ray inside the disk.

In the case of the two-dimensional system shown in Fig. C.1, $S(0)$ is given by

$$S(0) = \frac{k^2}{2\pi}\int_{-a}^{a}[1-e^{-i\rho\sin(\tau)}]dx, \tag{C.2}$$

where k is the angular wavenumber, a is the radius of the disk, $\tau = \cos^{-1}\left(\frac{x}{a}\right)$ is the angle as indicated in Fig. C.1 and $\rho = 2ka(m-1)$.

In the geometrical shadow below the disk, the field that is added to the original plane wave is the expression inside the brackets of Eq. C.2. The integral of $S(0)$ can be written in terms of Bessel functions, which then results in

$$Q_{ext}(\rho) = 2 - 2J_0(\rho) + 4\sum_{n=1}^{\infty} J_{2n}(\rho)\frac{1}{4n^2-1}, \tag{C.3}$$

where J_0 is the 0th order Bessel function of the first kind and J_{2n} are Bessel functions of the first kind and order $2n$.

In the Fig. 3a in the paper is Eq. C.3 compared with Mie theory.

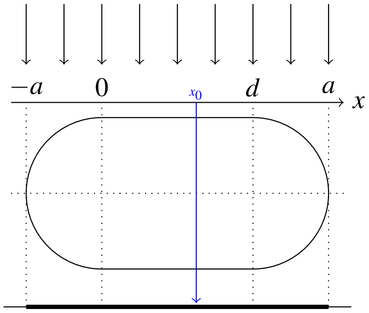

Figure C.2. System investigated for the evaluation of the van de Hulst approximation of the extinction efficiency of a stadium.

When we extend our circular scatterer to a stadium-shaped scatterer, as in Fig. C.2, we also need to include rays that are propagating through the rectangular mid-section of the stadium. The phase lag in this case is given by $2a(m-1)k$. The amplitude function in forward direction is given by

$$S_{stadium}(0) = \frac{k^2}{2\pi}\left(\int_{-a}^{0}[1-e^{-i\rho\sin(\tau)}]dx + \int_{0}^{d}[1-e^{-i\rho}]dx + \int_{d}^{d+a}[1-e^{-i\rho\sin(\tau)}]dx\right), \tag{C.4}$$

where k is the angular wavenumber and a and d are the radius of the stadium's end caps and the lengths of the straight sections as indicated in Fig. C.2.

With the result in Eq. C.4, we arrive at the following explicit formula for the extinction efficiency

$$Q_{ext}(\rho) = \frac{2a}{2a+d}\left(2-2J_0(\rho)+4\sum_{n=1}^{\infty}J_{2n}(\rho)\frac{1}{4n^2-1}\right)+\frac{2d}{2a+d}\left(1-\cos(\rho)\right). \tag{C.5}$$

In Fig. 3b, we compare the results of Eq. C.5 with electromagnetic COMSOL simulations.

D Electric near field of a stadium-shaped scatterer

Figure D.1 shows how the norm of the electric field inside a stadium-shaped scatterer changes as we increase the length, d, of the straight sections of the stadium (see Fig. 1d). The simulations are done by COMSOL Multiphysics. d is increased from 0 μm to 50 μm. The radius of the endcaps of the stadium (see Fig. 1d) is 10 μm. The refractive index of the scatterer is 1.8. The systems were investigated for a range of wavelengths, 4200 - 3700 $\frac{1}{\text{cm}}$, and plots in Fig. D.1 correspond to selected wavenumbers referring to peaks in $Q_{ext}(\tilde{\nu})$ for d less or equal to 2μm. The extinction efficiency $Q_{ext}(\tilde{\nu})$ is given in Fig. 3g. For small d, we observe the whispering gallery modes. On the way to a more deformed disk, we observe that for a weakly deformed disk the circular standing-wave pattern transforms into a diamond-shaped pattern (see e.g. D.1c and D.1g).

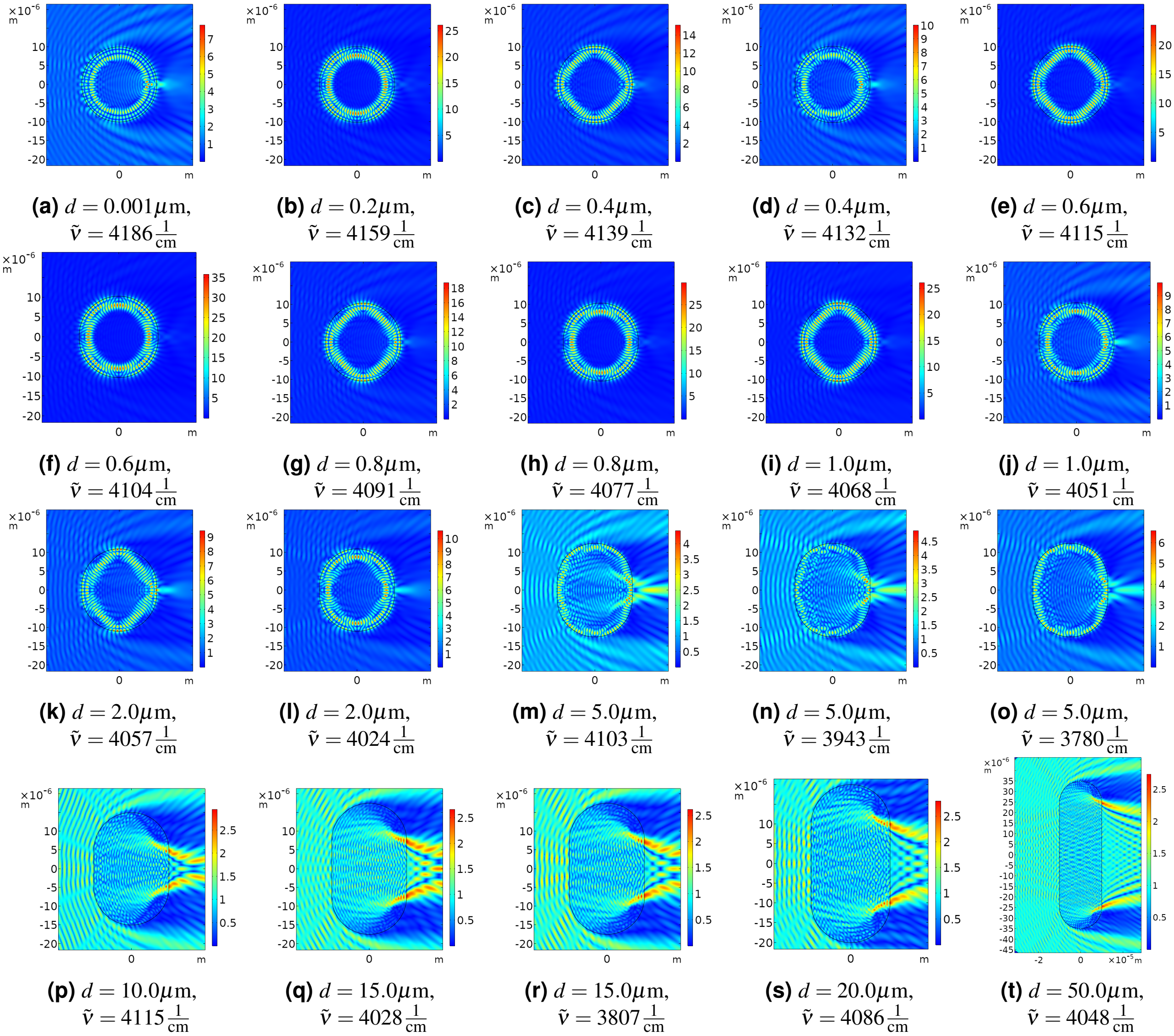

(a) $d = 0.001\mu\text{m}$, $\tilde{\nu} = 4186\frac{1}{\text{cm}}$
(b) $d = 0.2\mu\text{m}$, $\tilde{\nu} = 4159\frac{1}{\text{cm}}$
(c) $d = 0.4\mu\text{m}$, $\tilde{\nu} = 4139\frac{1}{\text{cm}}$
(d) $d = 0.4\mu\text{m}$, $\tilde{\nu} = 4132\frac{1}{\text{cm}}$
(e) $d = 0.6\mu\text{m}$, $\tilde{\nu} = 4115\frac{1}{\text{cm}}$
(f) $d = 0.6\mu\text{m}$, $\tilde{\nu} = 4104\frac{1}{\text{cm}}$
(g) $d = 0.8\mu\text{m}$, $\tilde{\nu} = 4091\frac{1}{\text{cm}}$
(h) $d = 0.8\mu\text{m}$, $\tilde{\nu} = 4077\frac{1}{\text{cm}}$
(i) $d = 1.0\mu\text{m}$, $\tilde{\nu} = 4068\frac{1}{\text{cm}}$
(j) $d = 1.0\mu\text{m}$, $\tilde{\nu} = 4051\frac{1}{\text{cm}}$
(k) $d = 2.0\mu\text{m}$, $\tilde{\nu} = 4057\frac{1}{\text{cm}}$
(l) $d = 2.0\mu\text{m}$, $\tilde{\nu} = 4024\frac{1}{\text{cm}}$
(m) $d = 5.0\mu\text{m}$, $\tilde{\nu} = 4103\frac{1}{\text{cm}}$
(n) $d = 5.0\mu\text{m}$, $\tilde{\nu} = 3943\frac{1}{\text{cm}}$
(o) $d = 5.0\mu\text{m}$, $\tilde{\nu} = 3780\frac{1}{\text{cm}}$
(p) $d = 10.0\mu\text{m}$, $\tilde{\nu} = 4115\frac{1}{\text{cm}}$
(q) $d = 15.0\mu\text{m}$, $\tilde{\nu} = 4028\frac{1}{\text{cm}}$
(r) $d = 15.0\mu\text{m}$, $\tilde{\nu} = 3807\frac{1}{\text{cm}}$
(s) $d = 20.0\mu\text{m}$, $\tilde{\nu} = 4086\frac{1}{\text{cm}}$
(t) $d = 50.0\mu\text{m}$, $\tilde{\nu} = 4048\frac{1}{\text{cm}}$

Figure D.1. The frames show the norm of the electric field for selected wavenumbers. An incoming plane wave with amplitude equal to 1 is propagating from left. The stadium-shaped scatterer has a radius of the end-caps equal to 10.0 μm and a refractive index of 1.8. The length, d, of the straight sections of the stadium and the associated selected wavenumbers are stated below each of the frames.

E Investigation of fractal behavior in path length plots

As discussed in the paper, life time plots were magnified seven times in order to investigate whether all of the generations exhibit fractal structures. The refractive index of the stadium shaped scatterer is 1.8. Classical ray tracing investigations are wavelength independent and the length of the straight sections of the stadium is 5 times the radius of the end caps. This corresponds to a system as shown in Fig. D.1t where $d = 50\mu$m and $r = 10\mu$m.

One tenth of the path length plot in Fig. 4b was magnified to the very left of the plot. The result is shown in Fig. E.1a. The figure shows two areas where the length of the ray is very sensitive to the start position, i.e we observe fractal structure in the life time plot. We magnified the left of these areas, i.e. the interval indicated by the red lines in Fig. E.1a. The result is shown in Fig.E.1b. We also observe a fractal structure in this plot. We further continued to magnify the very left of the 'brushes'. This procedure was repeated seven times. The corresponding path length plots are shown in Fig. E.1c-Fig. E.1g. The red lines indicate the magnified area. Fractal structure is present in all seven stages of magnification. The magnification corresponding factor of each of the generation plots is given in Tab. E.1. The magnifications also show that new structures appear at each stage of magnification. This indicates that the life-time fractal is not self-similar.

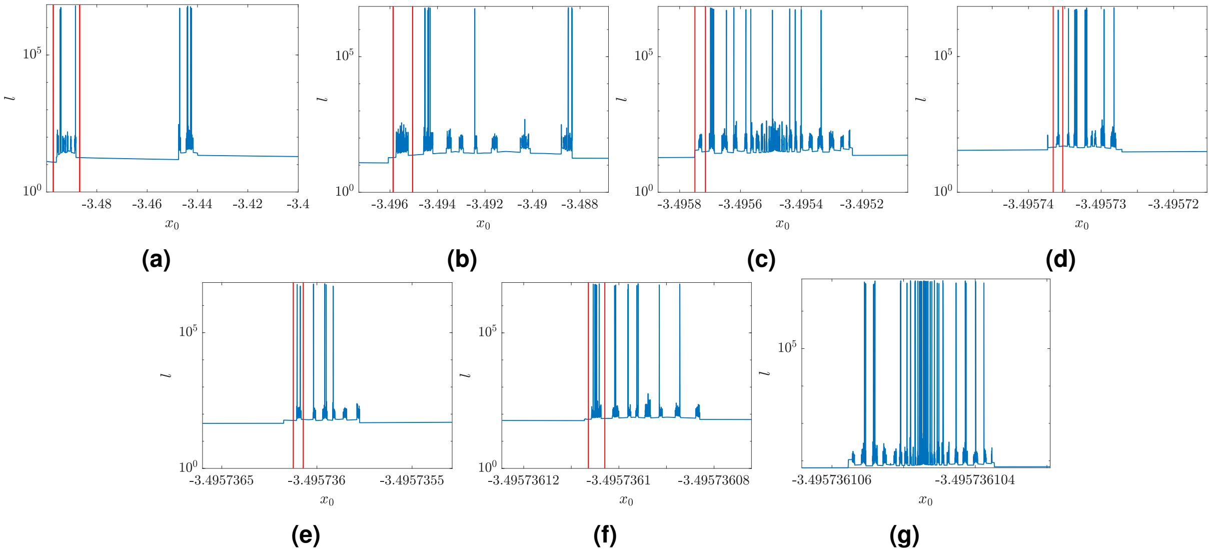

Figure E.1. The figures indicate the length of each ray as a function of start position. In frame (a) the start position is chosen to be a magnification of one tenth of the very left of Fig. 4b. Then the system is magnified seven times in the interval indicated by the two red lines. For each magnification, 100 000 rays are started. All the figures have a logarithmic y-axis. The refractive index of the system is 1.8 and the length of the straight section d is 5 times longer than the radius a (see Fig. 1d).

Table E.1. The table indicates the magnification factor for the generation plots in Fig. E.1.

Generation	Magnification factor
1	10
2	9.5
3	12.8
4	23.9
5	26.2
6	25.0
7	15.1

In order to further evaluate the fractal structure of the system, more subsets of the set of long-lived trajectories of the stadium system were magnified. In Fig. E.1. only the very left of the brushes was magnified in each generation. In the figures below, Fig. E.2, Fig. E.3 and Fig. E.4, all fractals in the two first generations are evaluated. As the figures below indicate, we have a spectrum of different fractal structures appearing at the different levels.

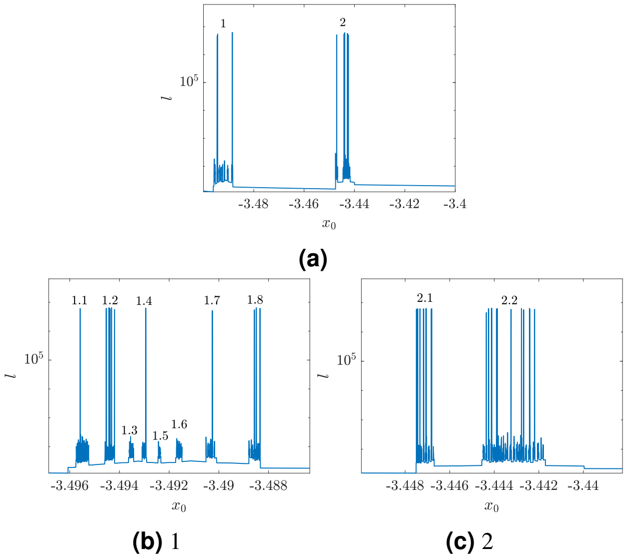

Figure E.2. Frame (a) is identical to Fig. E.1a and consists of two brushes, numbered from the left, **1** and **2**. Then each of these two brushes is magnified. Frame (b) shows the magnification of **1**, and frame (c) shows the magnification of **2**. As the figures show, the next generation of brushes is numbered from the left.

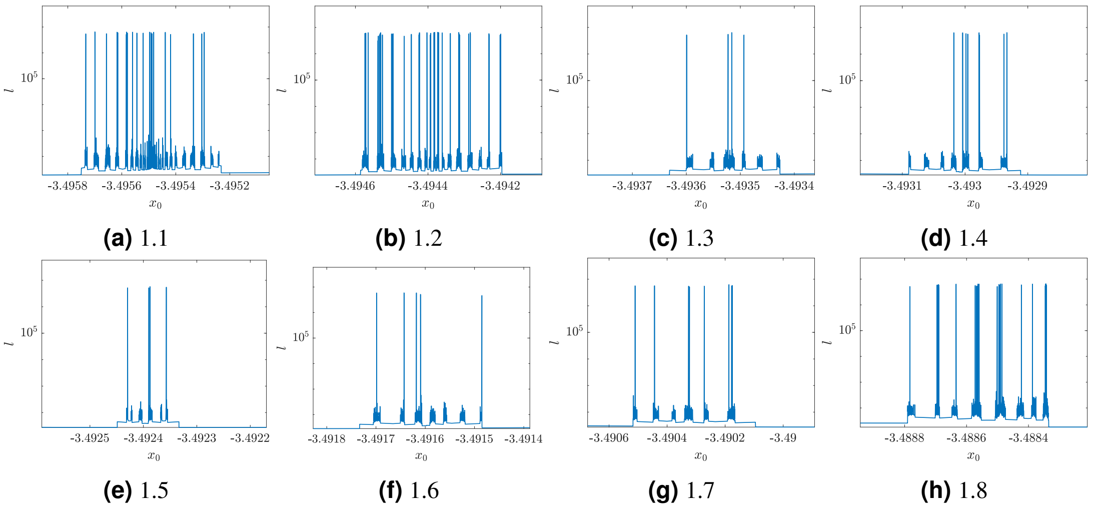

Figure E.3. Magnification of all fractal structures in Fig. E.2b. The sub-captions indicate which of the brushes in the previous generation a particular frame is associated with.

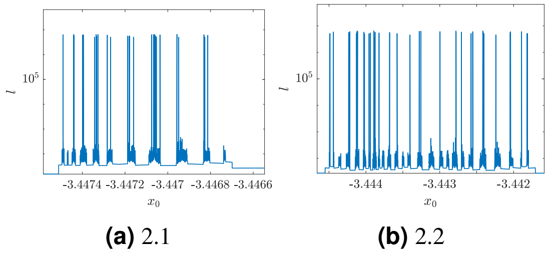

Figure E.4. Magnification of both fractal structures in Fig. E.2c. The sub-captions indicate which of the brushes in the previous generation a particular frame is associated with.

F Evaluation of the Lyapunov exponent

Several investigations were undertaken in order to evaluate the Lyapunov exponent of the system. As in Sec. E, the refractive index of the stadium-shaped scatterer is set to 1.8. We set the length of the straight sections of the stadium as 5 times the radius of the end caps (see Fig. 1d). As classical ray tracing investigations are wavelength independent, only the ratio of the straight sections of the stadium and the radius of the end caps affect the ray dynamics. The distances between pairs of long-lived rays from Fig. E.1g were evaluated.

The blue line of Fig. F.1a shows the distance between these two rays as a function of travelled path length. The logarithm of the distance between the two rays, D, is plotted against the distance travelled. The slope of the blue line is indicated by the red line, and indicates a Lyapunov exponent equal to 0.36. The start position of these two rays is indicated in Tab. F.1 as "Pair 1".

In order to evaluate the Lyapunov exponent for other starting positions, we evaluated the average of the Lyapunov exponent for 10 pair of rays (see Fig. F.1b). Fig. F.1c shows the average of the 10 pairs of rays of Fig. F.1b and is identical to the figure displayed in Fig. 4e. The starting positions of these 10 pairs of rays are given in Tab. F.1. The slope of the averaged line is found to be 0.36. The dips in the solid lines in Fig F.1 are due to the fact that the rays are crossing each other. The slope of the blue line in Fig F.1 (ignoring the dips) is the Lyapunov exponent. The red lines in Fig. F.1a and Fig. F.1c indicate that the slope is approximately 0.36.

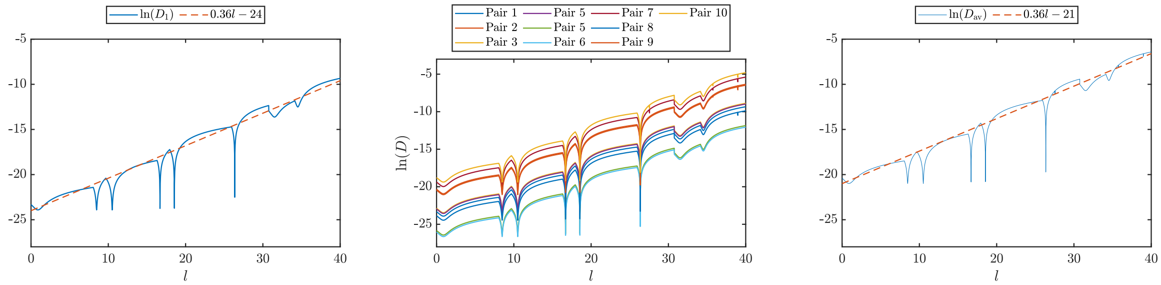

(a) (b) (c)

Figure F.1. Frame (a) The blue line is $\ln(D)$ where D is the distance between the two rays as a function of path length, l. The start position of the two rays is given as pair 1 in Tab. F.1. The red, dashed line indicates a straight line with the same trend as $\ln(D)$. The slope of this line, 0.36, is the Lyapunov exponent. Frame (b) shows $\ln(D)$ for 10 pairs of rays from Fig. E.1g. The start positions of the rays are given in Tab. F.1. Frame (c) shows the average of $\ln(D)$ for the 10 pairs of rays and the red, dashed line which indicates the slope, i.e. the Lyapunov exponent, which in this case 0.36.

Table F.1. The start position of the ten pairs of rays shown in Fig. F.1b. The ten pairs of rays are selected as neighbouring long-lived rays ($l \approx 10^5$) in Fig. E.1g.

	$x_{0,1}$	$x_{0,2}$
Pair 1	-3.495736105547581	-3.495736105546853
Pair 2	-3.495736105540718	-3.495736105530007
Pair 3	-3.495736105420616	-3.495736105420269
Pair 4	-3.495736105409732	-3.495736105409420
Pair 5	-3.495736105399923	-3.495736105399680
Pair 6	-3.495736105399368	-3.495736105399264
Pair 7	-3.495736105398918	-3.495736105398814
Pair 8	-3.495736105045579	-3.495736105043326
Pair 9	-3.495736105041593	-3.495736105041246
Pair 10	-3.495736104905339	-3.495736104904680

G Electric near field of an elliptic shaped scatterer

In the paper, the deformation of a disk into a ellipse was evaluated by simulations in COMSOL Multiphysics. Figure G.1 shows the near electric field for the ellipse shown in Fig. 5a. Figures G.1a-G.1c show the near field for selected wavenumbers which correspond to ripples in Fig. 5b for an elliptical scatterer of refractive index equal to 1.3. The a-parameter is kept constant at 10 μm and the b-parameter is increased from 10 to 60 μm (see Fig. 5a). The Figs. G.1d-G.1f correspond to the case where the refractive index is 1.8. The selected wavenumbers corresponds to ripples in Fig. 5c.

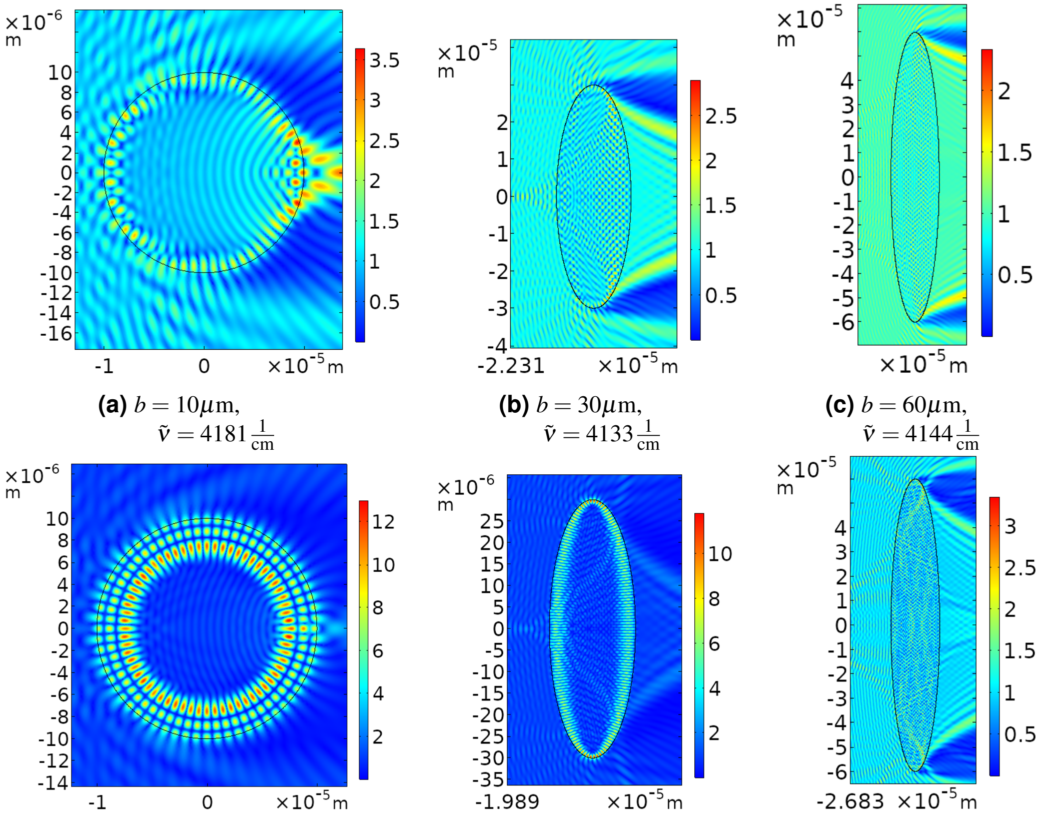

(a) $b = 10\mu$m, $\tilde{\nu} = 4181\,\frac{1}{\text{cm}}$

(b) $b = 30\mu$m, $\tilde{\nu} = 4133\,\frac{1}{\text{cm}}$

(c) $b = 60\mu$m, $\tilde{\nu} = 4144\,\frac{1}{\text{cm}}$

(d) $b = 10\mu$m, $\tilde{\nu} = 4086\,\frac{1}{\text{cm}}$

(e) $b = 30\mu$m, $\tilde{\nu} = 4115\,\frac{1}{\text{cm}}$

(f) $b = 60\mu$m, $\tilde{\nu} = 4052\,\frac{1}{\text{cm}}$

Figure G.1. The plots show the norm of the electric field for selected wavenumbers. The incoming wave is a plane wave entering from the left. The ellipse-shaped scatterer is shown in Fig. 5a. a is chosen as 10.0μm and the refractive index as 1.3 (panel a-c) and 1.8 (panel d-f), respectively. The parameter respective parameters b are shown in the sub-captions. The respective selected wavenumbers are shown in the sub-captions as well. The selected wavenumbers corresponds to ripples/peaks in Fig. 5b and Fig. 5c.

H The effect of a numerical aperture on Q_{sca}

In FTIR measurements the detector has a finite size, G, as indicated in Fig. 1a. The numerical aperture is related to the size of the detector as described in[3]. Due to the finite size of the detector, some of the scattered light hits the detector. From Mie theory the scattering cross section can be found over a selected interval[1]. The expressions for calculating Q_{sca} can be found in Supplementary Materials Sec. A.

The size of this detector is related to the *numerical aperture* , NA, of the FTIR spectrometer which is defined as

$$\mathrm{NA} = \sin(\theta), \tag{H.1}$$

where θ is the angle defined by the size of the detector as showed in Fig. H.1[3]. Figure H.1 is a simplified model of, e.g., a Schwarzschild optics for transmission IR measurements[4], that illustrates the general principle of how θ is related to NA.

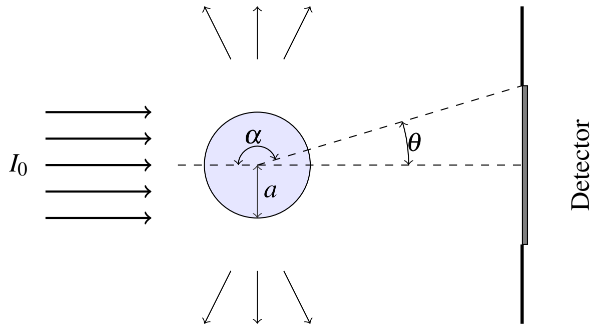

Figure H.1. Infrared light of intensity I_0 is propagating towards a sample with a shape of an infinite cylinder of radius a in an FTIR spectrometer. The detector of the spectrometer is of finite size, defined by the numerical aperture given by $\mathrm{NA} = \sin\theta$. The scattered light that does not hit the detector is limited within the angle α on both sides of the center line.

The extinction efficiency, Q_{ext}, is the sum of the scattering efficiency and the absorption efficiency. In the case of a non-absorptive scatterer we have Q_{ext} = Q_{sca}. In the case where the E-field is parallel to the cylinder axis, Q_{sca} can be found as a sum of b_n's as described in Eq. A.3. This expression is found by evaluating the following integral

$$Q_{sca} = \frac{1}{\pi ka}\int_0^{2\pi} |T(\theta)|^2 d\theta = \frac{2}{\pi ka}\int_0^{\pi} |T(\theta)|^2 d\theta, \tag{H.2}$$

where k is the angular wavenumber, a is the radius of the cylinder and θ is the angle as shown in Fig. H.1. The integral can be reduced as showed above due to symmetry. The function T in the case where the E-field is parallel to the cylinder axis is given by

$$T(\theta) = \sum_{n=-\infty}^{\infty} b_n e^{in\theta}, \tag{H.3}$$

where b_n is given in Eq.A.5.

When a numerical aperture is present, some of the scattered light is scattered in forward direction and hits the detector. In order to evaluate the amount of light which does not hit the detector, we need to evaluate how the Q_{sca} is affected by an increased θ. The scattering efficiency (Eq. H.2) is then evaluated over the angle α on both sides of the center line.

As mentioned above, the size of the numerical aperture determines the amount of scatted light that hits the detector. In order to evaluate how much of the light is extinguished from the forward direction, we evaluate the scattering efficiency of an infinite cylinder, where a plane wave is propagating perpendicular to the cylinder axis and the E-field is parallel to the cylinder axis. The investigation is done for a non-absorptive scatterer, i.e. $Q_{ext} = Q_{sca}$.

The extinction efficiency in the case where a numerical aperture is present is found by Eq. H.2, where the integral is taken over the angle α as described in Fig. H.1. The numerical apertures (NA) selected are NA= 0 (which corresponds to the exact expression for Q_{ext} (Eq. A.2)), NA= 0.325, and NA= 0.65[4,5]. The value of NA= 0.325 was chosen to lie midway between 0 and 0.65. Figure H.2 shows the result. The system investigated has a radius of 10 μm and a refractive index of 1.3 (Fig. H.2a) and 1.8 (Fig. H.2b).

As NA is increased, Q_{ext} decreases. This is expected, due to the reduced angle, α, that the integral is taken over. We see that both the ripples and their positions are preserved as NA increases.

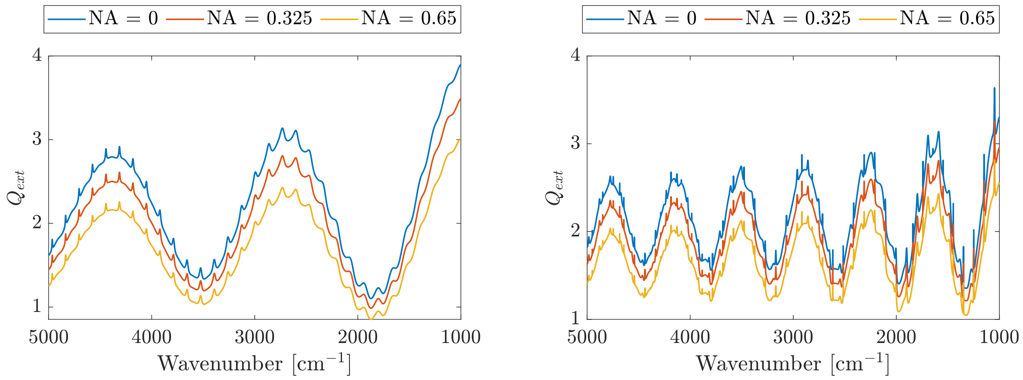

(a) (b)

Figure H.2. The extinction efficiency, which is equivalent to the scattering efficiency in the case of a non-absorptive circular scatterer, as found by Eq. H.2. The refractive index of the scatterer is (a) 1.3 and (b) 1.8, and the radius is 10 μm. As described in Fig. H.1, Q_{ext} is evaluated for the light that does not hit the detector with an angle larger than α. Q_{ext} is found for different sizes of the numerical aperture (NA). NA is related to θ in Fig. H.1 by Eq. H.1.

I The effect of absorption on Q_{ext}

In order to evaluate how absorption affects the extinction efficiency, we need to establish the connection between absorbance and the imaginary part of the refractive index. The absorption properties of a material can be modelled by including an imaginary part n_i in the refractive index, $m = n_r + in_i$. n_r is the real part and describes the refractive properties of the material. n_i can be related to the pure absorbance A_{pure} by

$$A_{pure} = \frac{4\pi n_i d_{\text{eff}} \tilde{\nu}}{\ln(10)}, \tag{I.1}$$

where d_{eff} is the effective thickness and $\tilde{\nu}$ is the wavenumber[6,7]. In the case of an infinite cylinder where the light is propagating perpendicular to the cylinder axis, $d_{\text{eff}} = \frac{\pi a}{2}$ where a is the radius of the cylinder. Figure I.1 shows how the absorbance is related to different choices of the imaginary part of the refractive index, n_i, within the wavenumber interval 5000-1000 cm^{-1}.

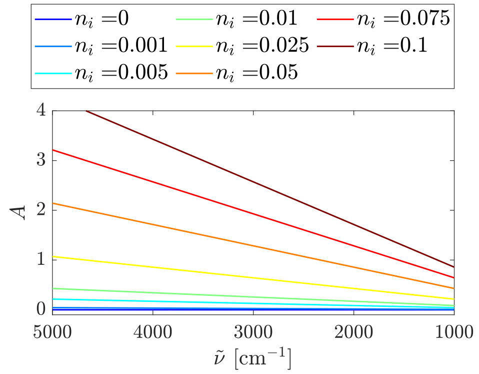

Figure I.1. The absorbance, A, as a function of wavenumber for different choices of the imaginary part of the refractive index, n_i. A is related to n_i as indicated by Eq. I.1. The effective thickness if found for a cylinder of radius 10 μm.

As mentioned in Supplementary Materials Sec. A, the extinction, scattering, and absorption efficiency for an infinite cylinder can be described exactly by Mie theory. By employing different values for the imaginary part of the refractive index in the equations for Q_{ext} (see Sec. A), Q_{ext} was evaluated for increasing absorbance values (see Fig. I.1).

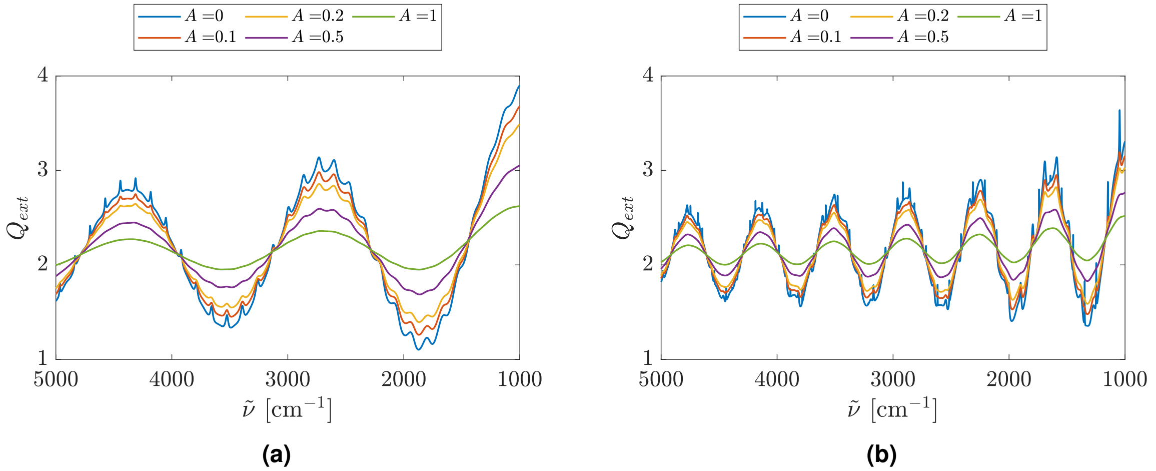

Figure I.2. The figure shows how Q_{ext} (Eq. A.2) changes as the absorbance of the sample increases. The radius of the sample is 10 μm and the real part of the refractive index is (a) 1.3 and (b) 1.8, respectively. The imaginary part of the refractive index is found by Eq. I.1.

Figure I.2 shows how Q_{ext} changes as the absorbance of the sample is increased. The absorbance is kept constant for all wavenumbers and is related to the imaginary part of the refractive index by Eq. I.1. The imaginary part of the refractive index, found from Eq. I.1 only explains the pure absorbance of the sample. In the case of a measurement (as shown in Fig. 2), the

apparent absorbance is found to be higher due to scattering effects. The real part of the refractive index in 1.3 and 1.8 in Fig. I.2a in Fig. I.2b, respectively. For both cases, the wiggle structure remains when increasing A, but the amplitudes of the wiggles decrease as A increases. In Fig. I.2b, the very sharp ripples disappear immediately when the absorbance is turned on. But the broader ripples are present until the the absorbance is equal to 0.5 for both cases.

Equation A.2 shows how Q_{ext} and Q_{sca} are composed of a sum of b_n's. For a cylinder, the ripples correspond to electric modes when the polarization is chosen along the axis of the cylinder. These electric modes are described by the b_n's in the Mie theory. The b_n are given in Eq. A.5, and by evaluating $\Re(b_n(\tilde{\nu}))$, how the ripples in Q_{ext} correspond to peaks $\Re(b_n(\tilde{\nu}))$. The different ripples corresponds to peaks in b_n for different n's.

Figure I.3 shows Q_{ext} (Eq. A.2), black line, and b_{15} (Eq. A.5), blue line, for increasing absorbance for a disk of radius 10 μm and a real part of the refractive index equal to 1.8. The first sharp peak from the right in b_{15} in Fig I.3a at $\tilde{\nu} = 1643.5 \frac{1}{\text{cm}}$ corresponds to a sharp ripple in Q_{ext}. The peak corresponds to a whispering gallery mode of first order. The second peak in b_{15} from the right is at $\tilde{\nu} = 2003.3 \frac{1}{\text{cm}}$ and corresponds to a broader peak in Q_{ext}. This peak is a whispering gallery mode of second order. The wave function of the two ripples are shown in Fig. I.4. Notice the scale on the color bar.

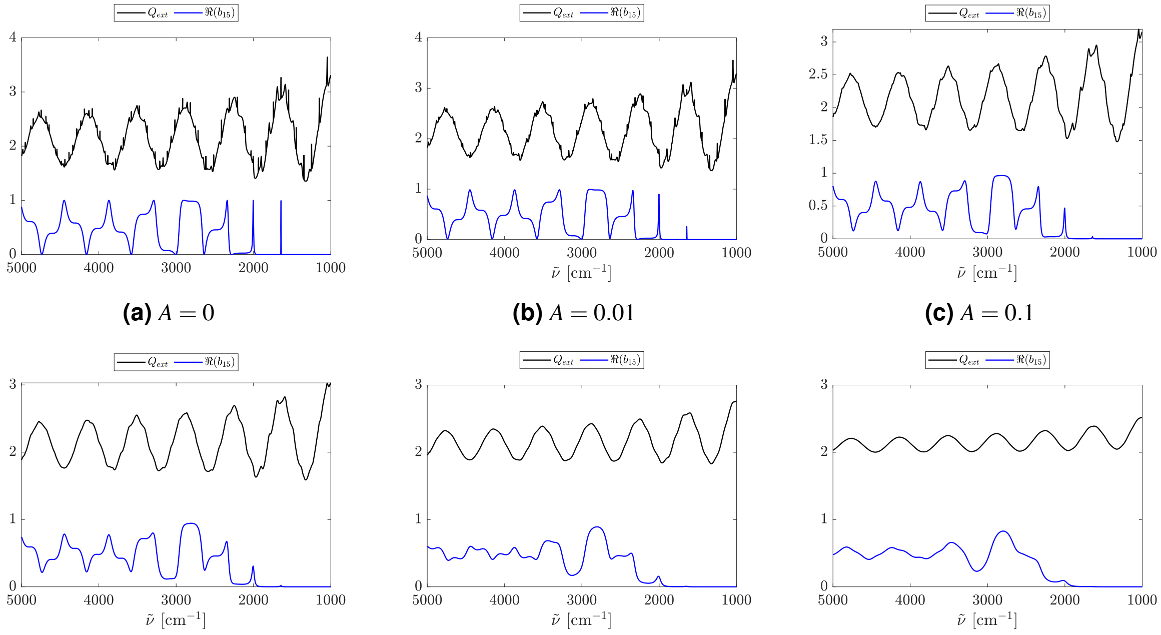

(a) $A = 0$ **(b)** $A = 0.01$ **(c)** $A = 0.1$

(d) $A = 0.2$ **(e)** $A = 0.5$ **(f)** $A = 1$

Figure I.3. The frames show how Q_{ext} (black line) (Eq. A.2) and b_{15} (blue line) (Eq. A.5) are changing as the absorbance (A) is increased. The radius of the cylinder is 10 μm, and the refractive index is given by $m = n_r + in_i$, where n_r = 1.8, and n_i is related to A (given in the respective sub-captions) by Eq. I.1.

Figure I.5 shows Q_{ext} (Eq. A.2), black line, and b_{20} (Eq. A.5), blue line, for increasing absorbance for a disk of radius 10 μm and a real part of the refractive index equal to 1.3. The first sharp peak from the right in b_{20} in Fig I.5a at $\tilde{\nu} = 2865.1 \frac{1}{\text{cm}}$ corresponds to a ripple in Q_{ext}. In Fig. I.5b is $Q_{ext}(\tilde{\nu})$ plotted in a smaller wavenumber interval with a very fine resolution in order to look for a needle-sharp resonance to the right of the resonance at $\tilde{\nu} = 2865.1 \frac{1}{\text{cm}}$. No peak is observed. The peak at $\tilde{\nu} = 2865.1 \frac{1}{\text{cm}}$ corresponds to a whispering gallery mode of first order. The wave function of the ripple is shown in Fig. I.6. Notice the scale on the color bar.

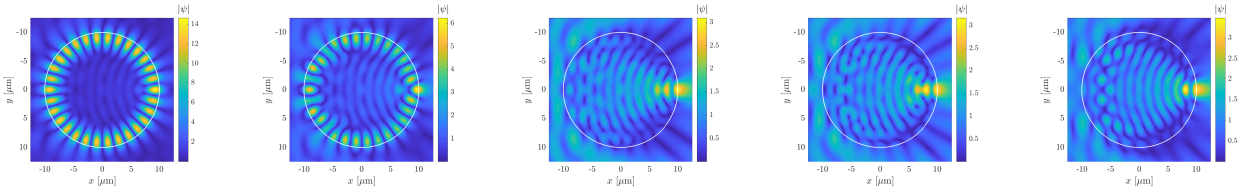

(a) $A = 0$, $\tilde{\nu} = 1643.5\ \frac{1}{\text{cm}}$ **(b)** $A = 0.01$, $\tilde{\nu} = 1643.5\ \frac{1}{\text{cm}}$ **(c)** $A = 0.1$, $\tilde{\nu} = 1643.5\ \frac{1}{\text{cm}}$ **(d)** $A = 0.5$, $\tilde{\nu} = 1643.5\ \frac{1}{\text{cm}}$ **(e)** $A = 1$, $\tilde{\nu} = 1643.5\ \frac{1}{\text{cm}}$

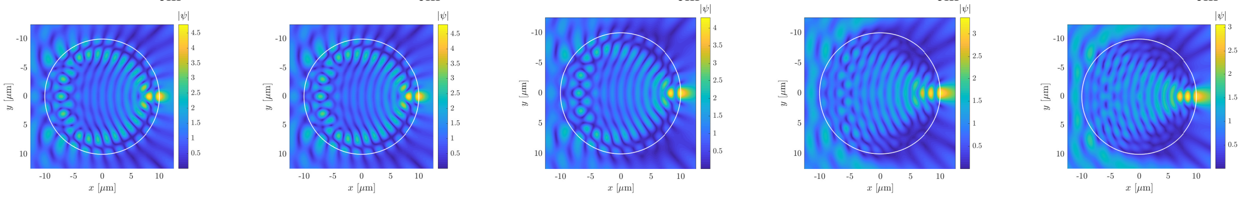

(f) $A = 0$, $\tilde{\nu} = 2003.3\ \frac{1}{\text{cm}}$ **(g)** $A = 0.01$, $\tilde{\nu} = 2003.3\ \frac{1}{\text{cm}}$ **(h)** $A = 0.1$, $\tilde{\nu} = 2003.3\ \frac{1}{\text{cm}}$ **(i)** $A = 0.5$, $\tilde{\nu} = 2003.3\ \frac{1}{\text{cm}}$ **(j)** $A = 1$, $\tilde{\nu} = 2003.3\ \frac{1}{\text{cm}}$

Figure I.4. The frames show the norm of the wave function, i.e. the electric field, for the first two ripple structures contained in b_{15}. The wave function is calculated as described in Supplementary Materials Sec. A. The incident plane wave is propagating from the left with a wavelength corresponding to the wavenumber in the respective sub-captions of each frame. The real part of the refractive index of the circular scatterer is 1.8 and the imaginary part is found by Eq. I.1 from the absorbance given in the respective sub-captions. The radius of the scatterer is 10 μm. The color bar is not kept constant.

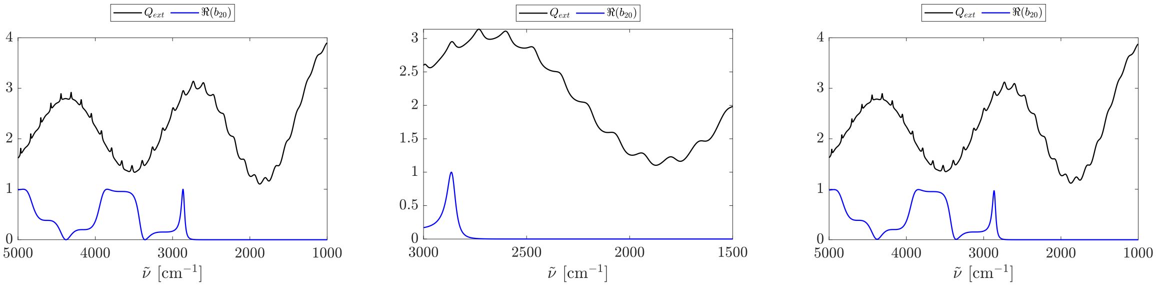

(a) $A = 0$ **(b)** $A = 0$ and $\delta\tilde{\nu} = 0.0001\text{cm}^{-1}$ **(c)** $A = 0.01$

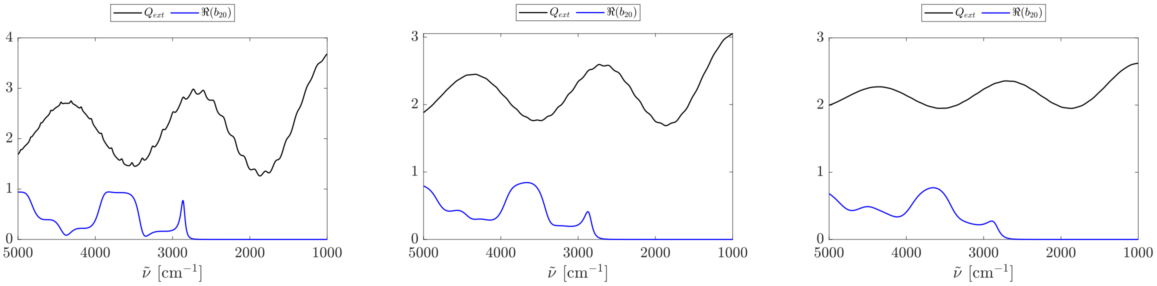

(d) $A = 0.1$ **(e)** $A = 0.5$ **(f)** $A = 1$

Figure I.5. The frames show how Q_{ext} (black line) (Eq. A.2) and b_{15} (blue line) (Eq. A.5) are changing as the absorbance (A) is increased. The radius of the cylinder is 10 μm, and the refractive index is given by $m = n_r + in_i$ where n_r = 1.3 and n_i is related to A (given in the respective sub-captions) by Eq. I.1. Frame (b) shows the behavior of Q_{ext} in a smaller wavenumber interval with a very fine resolution. A needle-sharp resonance to the right of the resonance at $\tilde{\nu} = 2865.1\frac{1}{\text{cm}}$ is not observed.

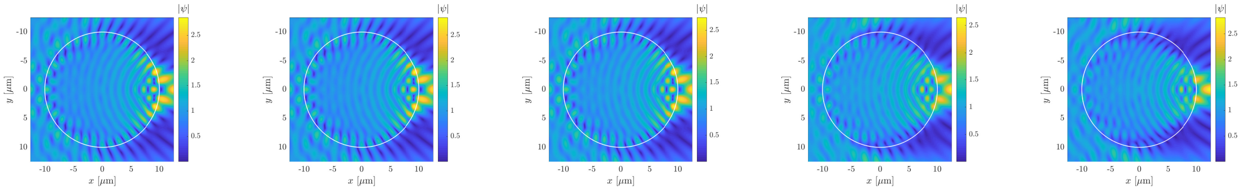

(a) $A = 0$, $\tilde{\nu} = 2865.1\ \frac{1}{\text{cm}}$

(b) $A = 0.01$, $\tilde{\nu} = 2865.1\ \frac{1}{\text{cm}}$

(c) $A = 0.1$, $\tilde{\nu} = 2865.1\ \frac{1}{\text{cm}}$

(d) $A = 0.1$, $\tilde{\nu} = 2865.1\ \frac{1}{\text{cm}}$

(e) $A = 1$, $\tilde{\nu} = 2865.1\ \frac{1}{\text{cm}}$

Figure I.6. The frames show the norm of the wave function, i.e., the electric field, for the first ripple structure contained in b_{20}. The wave function is calculated as described in Supplementary Materials Sec. A. The plane wave is incident from the left with wavenumber $\tilde{\nu} = 2865.1\frac{1}{\text{cm}}$. The real part of the refractive index of the circular scatterer is 1.3 and the imaginary part is found by Eq. I.1 from the absorbance given in the respective sub-captions. The radius of the scatterer is 10μm. The color bar is not kept constant.

References

1. Hulst, H. C. *Light Scattering by Small Particles* (Dover Publications, Inc., 1981).
2. Mie, G. Contribution to the optical properties of turbid media, in particular of colloidal suspensions of metals. *Ann. Phys.(Leipzig)* **25**, 377–452 (1908).
3. Tipler, P. A. & Mosca, G. *Physics for scientists and engineers : with modern physics* (Freeman, New York, 2008), 6th ed. edn.
4. Van Dijk, T., Mayerich, D., Carney, P. S. & Bhargava, R. Recovery of absorption spectra from fourier transform infrared (ft-ir) microspectroscopic measurements of intact spheres. *Appl. Spectrosc.* **67**, 546–552 (2013).
5. Bhargava, R. Infrared spectroscopic imaging: the next generation. *Appl. spectroscopy* **66**, 1091–1120 (2012).
6. Konevskikh, T., Lukacs, R., Blümel, R., Ponossov, A. & Kohler, A. Mie scatter corrections in single cell infrared microspectroscopy. *Faraday Discuss.* **187**, 235–257 (2016).
7. Blümel, R., Lukacz, R., Zimmermann, B., Bağcioğlu, M. & Kohler, A. Observation of Mie ripples in the synchrotron Fourier transform infrared spectra of spheroidal pollen grains. *J. Opt. Soc. Am. A* **35**, 1769–1779 (2018).
